# Two unreported lecanoric acid derivatives isolated from *Chaetomium globosum* KM651986 and their anti-diabetic effect

**DOI:** 10.1038/s41598-025-05875-4

**Published:** 2025-07-02

**Authors:** Rehab A. Hussein, Enaam M. AbouZeid, Dalia O. Saleh, Gehad A. Abdel Jaleel, Abeer A. Abd El Aty, Hiroyuki Morita, Ahmed A. El-Beih

**Affiliations:** 1 Pharmacognosy Department, Pharmaceutical and Drug Industries Research Institute, National Research Centre, Dokki, 12622 Giza Egypt; 2 Pharmacology Department, Medical Research and Clinical Studies Institute, National Research Centre, Dokki, 12622 Giza Egypt; 3 Chemistry of Natural and Microbial Products Department, Pharmaceutical and Drug Industries Research Institute, National Research Centre, Dokki, 12622 Giza Egypt; 4 Institute of Natural Medicine, University of Toyama, 2630‑Sugitani, Toyama, 930‑0194 Japan

**Keywords:** *Chaetomium globosum*, SwissTargetPrediction, Lecanoric acid derivatives, Anti-diabetic, Glucose-6-phosphate dehydrogenase, Biochemistry, Biotechnology, Computational biology and bioinformatics, Drug discovery, Microbiology, Endocrinology

## Abstract

The genus *Chaetomium*, Chaetomiaceae, is a promising source of secondary metabolites with proven pharmacological activities. In the present study, a new strain of *Chaetomium globosum* (KM651986) was investigated resulting in the isolation of two unreported lecanoric acid derivatives **1** and **2**, for the first time, which were analyzed using spectroscopic methods, SwissSimilarity, and SwissTargetPrediction. Thus, predicting the possible biological targets for **1** and **2.** Consequently, the crude methanol extract of *C. globosum*, compounds **1** and **2** were investigated for their anti-diabetic activity using streptozocin (STZ) - induced diabetic rat model. Results indicated that the crude extract *C. globosum* and compounds **1** and **2** significantly impacted serum glucose, insulin, HbA1c levels, hepatic glucose-6-phosphate dehydrogenase (G6PD) content, cAMP levels, and hepatic α-amylase content. Compound **2** showed a significant decrease in serum glucose by 40% and an increase in insulin level by 65% compared to STZ-diabetic rats, indicating its potent anti-hyperglycemic effect and potential in improving insulin secretion and cell regeneration. Compound **2** also elevated hepatic G6PD, normalized cAMP levels, and decreased hepatic α-amylase, indicating its potential in improving glucose metabolism at the hepatic level. Therefore, compound **2** emerges as particularly promising anti-diabetic candidate meriting further investigation into its potential therapeutic mechanisms of action.

**Supplementary Information:**

The online version contains supplementary material available at 10.1038/s41598-025-05875-4.

## Introduction

The dematiaceous filamentous fungus, *Chaetomium globosum*, Chaetomiaceae, belongs to the largest fungal phylum Ascomycota (sac fungi)^1^. The genus *Chaetomium* comprises more than 400 species which are distributed worldwide in the soil, atmosphere, food, plant debris and even in the sediments of marine mudflats. Species belonging to *Chaetomium* are pathogenic such as *C. globosum*, *C. strumarium* and *C. atrobrunneum* causing opportunistic infections that ranges from onychomycosis, sinusitis, and cutaneous and subcutaneous mycoses to fatal cerebral disease and pneumonia to according to the individual’s immunity status^2–4^. While others are of economic importance for their utilization in industry such as *C. cochliodes* that produces the textile pigment cochlidinol, or as a biofuel source e.g. *C. elegans*,* C. variabile* and *C. fusiforme*^2^. Moreover, *Chaetomium* spp. are considered as a potential source of secondary metabolites that have proven pharmacological activities.

Chaetoglobol acid from *C. globosum* exhibited potent inhibition of both α-glucosidase and α-amylase in vitro. Cytochalasans from *C. globosum* exhibit potent antitumor activity by disrupting actin polymerization, inducing G2/M cell cycle arrest, and triggering apoptosis via the PI3K/Akt/mTOR pathway^5^. Indole alkaloids, such as chaetoglobinols from *C. elatum*, act as competitive α-glucosidase inhibitors, offering therapeutic potential for diabetes management by delaying carbohydrate digestion^6^. Additionally, azaphilones (e.g., chaetofusins from *C. fusiforme*) display broad-spectrum antifungal activity by interfering with ergosterol biosynthesis, a critical component of fungal cell membranes. Beyond these, *Chaetomium* yields diverse metabolites—including chaetoglobosins, epipolythiodioxopiperazines, xanthones, and terpenoids—that contribute to its pharmacological and biotechnological potential. Consequently, research on *Chaetomium* is nevertheless continuous to discover more about this unique fungus. SwissSimilarity and SwissTargetPrediction are major bioinformatics projects by the Swiss Institute of Bioinformatics (SIB) offering online resources for computer-aided drug design and chemical biology research. SwissSimilarity identifies structurally similar molecules by virtually screening several chemical libraries whereas SwissTargetPrediction predicts biological targets for a molecule^7,8^. SwissSimilarity molecular output list is prioritized with compounds that are expected to share protein targets with that query, thus could be investigated experimentally. Several libraries are accessible for screening using SwissSimilarity including approved medications, clinical candidates, pharmacologically active molecules, as well as synthetic and commercial compounds. 2D and 3D molecular descriptors, including path-based FP2 fingerprints and ElectroShape vectors, were used in the initial version of SwissSimilarity, which was introduced in 2015. However, novel fingerprinting techniques for molecular description have been developed in recent years. The new version of the SwissSimilarity includes additional 2D and 3D approaches for molecular similarity estimation, extended-connectivity, MinHash, 2D pharmacophore, extended reduced graph, and extended 3D fingerprints. The interface of the website has been comprehensively rebuilt to provide a better user experience with the updated version accessible for free since December 2021^7^. On the other hand, SwissTargetPrediction combines 2D and 3D similarity parameters to accurately predict targets of bioactive molecules. Five distinct organisms can be used to test predictions and close paralogs and orthologs map predictions by homology both within and between species^8^.

Two unreported lecanoric acid derivatives were isolated for the first time from the newly identified strain *Chaetomium globosum* KM651986. After complete spectral analysis and structural elucidation, a similarity check for both compounds was performed using SwissSimilarity tool. Similarly, SwissTargetPrediction identified the possible biological targets of the two isolated compounds. Consequently, the two compounds as well as the crude extract of *Chaetomium globosum* were investigated for their anti-diabetic activity using streptozocin (STZ)-induced diabetic rat model.

## Methods

### Preparation of the extracts

#### Isolation and identification of fungi

The fungal isolate *Chaetomium globosum* was isolated from shrimp shell wastes^8^. Various shrimp shell wastes were gathered in sterilized plastic bags, then washed with distilled water, wiped between filter paper and placed in Czapek’s Dox agar, treated with agar, under sterile conditions to inhibit bacteria. *Chaetomium globosum* was isolated from the plates by incubating at 28 °C for up to 10 days, then these fungal isolates were kept at 4 °C on potato dextrose agar (PDA) slants in the Chemistry of Natural and Microbial Products Department’s microbiology lab, NRC. The National Research Center’s Department of Chemistry of Natural and Microbial Products’ Microbial Culture Collection Unit (MCCU) identified the pure fungal isolate by comparing its microscopic and colonial characteristics with morphological characterization of previously recognized fungal species^9^.

The preliminary identification of fungal isolate was performed on basis of their distinctive morphological and microscopic characteristics, particularly the production of globose to ovate ascomata with hair-like structures. Ascospores were Lemon-shaped (limoniform), with septated and branched hyphae. On culture media the growth rate was moderate to fast and fungal colony appeared with Woolly to cottony texture with white color turning to gray with age. The reverse of plate typically dark due to pigment production.

**Fig. 1:**
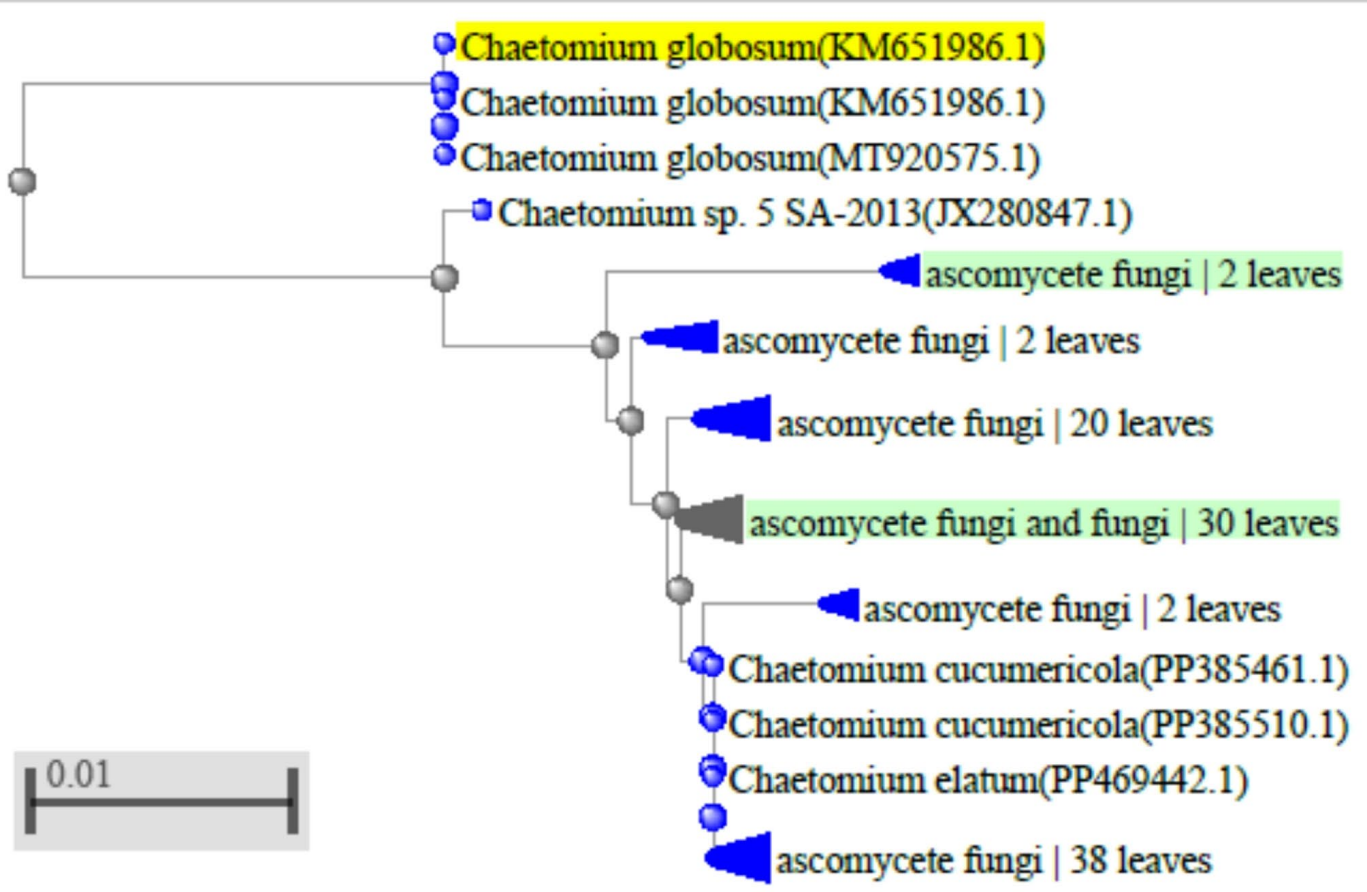
The phylogenetic tree based on partial sequencing of 18 S rDNA gene showing relationship neighbour-joining between the fungal isolate *Chaetomium globosum* KM651986 and other closely related sequences on NCBI Gen Bank reference taxa.

For accurate identification and confirmation of the morphologically identified strain, the most reliable molecular typing technique based on partial sequencing and analysis of the 18 S rDNA was used. Sequence analysis of 18 S rDNA of the fungal isolate with available NCBI Gen Bank database showed high identity (100%) to *Chaetomium globosum* isolate O322B (Gene Bank accession number *MT920575*), and revealed a close similarity (94%) with *Chaetomium globosum* strain c2 (Gene Bank accession number *KX011855*), and *Chaetomium globosum* strain YIMPH30021 (Gene Bank accession number *KP230822*), as shown in the phylogenetic tree (Fig. 1).

Finally, data derived from morphology, and phylogenetic analyses identified this fungus as ***Chaetomium globosum*** and deposited in the Gen Bank under accession number KM651986.

### Preparation of fungal extract

*Chaetomium globosum* KM651986 was cultivated on agar medium (20 ml X 180 plates) composed of 2.0% malt extract and 5.0% peptone in 1 L distilled water. After incubation at 25 ºC for 20 days, the cultures were extracted thrice with MeOH (5 L). The MeOH extract was evaporated to dryness under reduced pressure to obtain a dark brown gum. The crude MeOH extract was partitioned between EtOAc and distilled water for three successive times to give EtOAc fraction (3.5 g).

#### Isolation of compounds 1 and 2

The EtOAc fraction was subjected to Silica Gel column chromatography and eluted with CHCl_3_/MeOH (15:1, 9:1 and 4:1) to yield 9 fractions (A1–A9). Fraction A3 was subjected to ODS column chromatography using MeOH (60–90%) to yield 6 fractions (B1-B6). Subfraction B3 was further purified with ODS HPLC column, 70% MeOH to afford **2** (19 mg). Subfraction B4 was purified with ODS HPLC using 50% MeOH to afford **1** (7 mg).

#### Spectral analysis of compounds 1 and 2

**Fig. 2:**
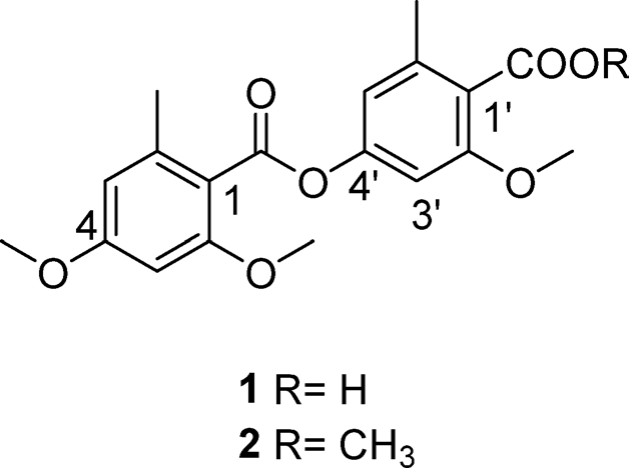
Structures of compounds 1 and 2.

**Fig. 3:**
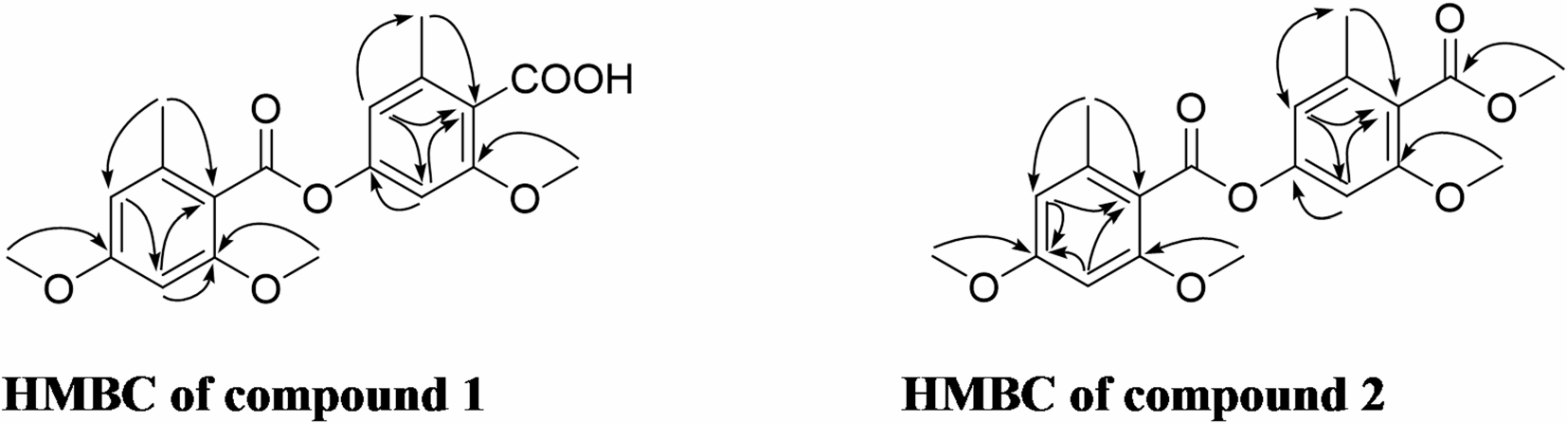
HMBC of compounds 1 and 2.

Compound **1** was isolated as yellow gum. The HRESI reported a molecular ion peak [M-H]^−^ *m/z* 359.1130 representing the molecular formula C_19_H_20_O_7_. The IR spectrum shows 2 absorption bands at 1765 and 1729 confirming the presence of two carboxylic group. The^1^H NMR spectrum (Table 1) showed two singlet methyl groups signals (*δ*_H_ 2.53, 6-CH_3_ and*δ*_H_ 2.44, 6′-CH_3_), three singlet methoxy groups signals (*δ*_H_ 3,84, 2-OCH_3_, *δ*_H_ 3.95, 4-OCH_3_ and *δ*_H_ 3.87, 2′-OCH_3_), four aromatic protons signals, each two were metacoupled confirming the presence of two aromatic rings (*δ*_H_ 6.76, d, *J* = 1.6 Hz, H-3, *δ*_H_ 6.78, d, *J* = 1.6 Hz, H-5, *δ*_H_ 6.38, d, *J* = 1.6 Hz, H-3′ and *δ*_H_ 6.39, d, *J* = 1.6 Hz, H-5′). The ^13^C NMR spectrum (Table 1) displayed 19 carbons, including two methyl carbons (*δ*_C_ 21.3, 6-CH_3_ and *δ*_C_ 20.3, 6′-CH_3_), four methine aromatic carbons (*δ*_C_ 103.2, C-3, *δ*_C_ 117.1, C-5, *δ*_C_ 96.4, C-3′ and *δ*_C_ 107.1, C-5′), two carbonyl carbons (*δ*_C_ 166.0, 1-COO and *δ*_C_ 168.3, 1′-COO), three methoxy carbons (*δ*_C_ 55.4, 2-OCH_3_, *δ*_C_ 55.6, 4-OCH_3_ and *δ*_C_ 56.1 2′-OCH_3_), and eight quaternary-aromatic carbons including four oxygenated carbons (*δ*_C_ 158.4, C-2, *δ*_C_ 153.3, C-4, *δ*_C_ 159.0, C-2′, and *δ*_C_ 162.1, C-4′) and four non-oxygenated carbons (*δ*_C_ 118.1, C-1, *δ*_C_ 141.8, C-6, *δ*_C_ 114.8, C-1′ and *δ*_C_ 139.2, C-6′). From ^1^H to ^13^C NMR and analysis of the 2D NMR spectra, compound **1** was deduced to have the depside skeleton similar to lecanoric acid. The methoxy groups at positions 2, 4 and 2′ were confirmed by HMBC correlations with carbons 2, 4 and 2′ respectively. The two methyl groups at positions 6 and 6′ were confirmed by HMBC correlations with C-1, C-5 and C-1′ and C-5′, respectively (Fig. 3). Thus compound **1** was identified as 4-[(2,4-Dimethoxy-6-methylbenzoyl)oxy]-2-methoxy-6-methylbenzoic acid (Fig. 2).

Similarly, compound **2** was isolated as yellow gum. The NMR spectra of compound **2** shows a great similarity to compound **1.** The only difference is the appearance of an extra methoxy group (*δ*_H_ 3.91, s, *δ*_C_ 52.2). The position of this methoxy group was confirmed by the HMBC correlation to the carboxylic carbon 1′-COO (*δ*_C_ 168.3) (Fig. 3). This was also confirmed from the HRESI mass spectrum by the appearance of the molecular ion peak [M + H]^+^ 375.1446 representing the molecular formula C_20_H_22_O_6_. Thus compound **2** was identified as 3-methoxy-4-(methoxycarbonyl)-5-methylphenyl 2,4-dimethoxy-6-methylbenzoate (Fig. 2).

Compounds **1** and **2** are reported for the first time from fungi. Compound **2** have been previously reported from lichens with only ^13^C NMR data^10^.

**Table 1:** ^1^H and ^13^C NMR data for 1 and 2 in CDCl_3_ (*J* in Hz).

Position	1			2		
	δ_H_ (Hz)	δ_C_		δ_H_ (Hz)	δ_C_	
1		118.0,	C		115.1,	C
2		158.4,	C		158.8,	C
3	6.76, d (1.6)	103.2,	CH	6.36, d (2.0)	96.3,	CH
4		153.3,	C		161.9,	C
5	6.78, d (1.6)	117.1,	CH	6.37, d (2.0)	106.9,	CH
6		141.8,	C		139.0,	C
1′		114.8,	C		121.2,	C
2′		159.0,	C		137.8,	C
3′	6.38, d (1.6)	96.4,	CH	6.69, d (2.0)	115.4,	CH
4′		162.1,	C		152.4,	C
5′	6.39, d (1.6)	107.1,	CH	6.64, d (2.0)	102.8,	CH
6′		139.2,	C		157.5,	C
2-OCH_3_	3.84, s	55.4,	CH_3_	3.86, s	56.1,	CH_3_
4-OCH_3_	3.95, s	55.6,	CH_3_	3.83, s	55.4,	CH_3_
6-CH_3_	2.53, S	21.3,	CH_3_	2.43, s	20.2,	CH_3_
1′-COOR		168.5,	C		168.3,	C
2′-OCH_3_	3.87, s	56.1,	CH_3_	3.83, s	56.0,	CH_3_
6′-CH_3_	2.44, s	20.3,	CH_3_	2.31, s	19.5,	CH_3_
1-COO		166.0,	C		166.1,	C
1′-COOCH_3_				3.91, s	52.2,	CH_3_

### Virtual similarity screening and target prediction for compounds 1 and 2

Compounds **1** and **2** were virtually screened for structural similarity using the SwissSimilarity tool against bioactive compounds from the Ligand Expo database. The analysis employed both 2D and 3D similarity metrics to identify structurally related compounds. To predict potential biological targets, the SwissTargetPrediction platform was used, which integrates 2D and 3D molecular similarity measures into a Combined-Score. A score above 0.5 indicates a high likelihood of shared protein targets between the query compounds and known bioactives. Detailed methodology is provided in the Supplementary Information^7,8,11^. Detailed methodology are provided in the Supplementary file (S13).

### Animal study

#### Laboratory animals

Adult male Wistar albino rats weighing 150–200 g were employed for in vivo studies. The National Research Center’s animal house colony provided the animals. They were kept in plastic cages with standard lighting and dark cycles, a temperature of 25 ± 1 °C, a relative humidity of 55 ± 10%, and a standard pellet feed and ad libitum water. All procedures were conducted in compliance with the relevant ethical guidelines and regulations and in accordance with the ARRIVE guidelines and received approval from the Institutional Animal Ethics Committee (IAEC) (Approval number: 19039). Rats were anesthetized via intraperitoneal injection of ketamine (80 mg/kg) combined with xylazine (10 mg/kg). The depth of anesthesia was verified by checking for the absence of the pedal reflex and monitoring respiratory rate. Anesthesia was maintained throughout the procedure, with animals carefully observed for any signs of pain or distress. At the conclusion of the experimental period, euthanasia was performed using an overdose of sodium pentobarbital (200 mg/kg, i.p.). Death was confirmed by the cessation of vital signs, including respiratory and cardiac activity. These procedures were designed to minimize animal suffering and followed ethical standards for the humane treatment of laboratory animals.

#### Chemicals

Sigma-Aldrich (St. Louis, MO, USA) supplied the streptozotocin (STZ) (265.22 g/mol, purity ≥ 95%). The standard drug, metformin (MET) (129.16 g/mol, pharmaceutical grade), was purchased from Servier Canada (Laval, QC, Canada). All additional reagents and solvents were of analytical grade (purity ≥ 95–99%), as supplied by ADWIC (Egypt).

#### Acute oral toxicity study

An acute toxicity study was conducted for the crude methanol extract of *Chaetomium globosum* (KM651986) in accordance with the Organization for Economic Cooperation and Development (OECD) (Paris, France) Guideline 423 (Acute Toxic Class Method). Female Wistar rats (*n* = 6) were administered a single oral dose of 2000 mg/kg of the extract and observed continuously for 24 h and then daily for 14 days for any signs of toxicity, behavioral changes, or mortality. No adverse effects or deaths were recorded during the observation period, indicating the extract’s safety at this dose. Based on these findings, a dose of 100 and 200 mg/kg (1/10th and 1/20th of the safe dose) was selected for the in vivo anti-diabetic study.

#### Induction of diabetes mellitus

A single intraperitoneal (i.p.) dose of STZ (55 mg/kg) was used to induce diabetes mellitus. STZ diluted in a citrate buffer (0.1 M, pH 4.5) was administered into rats after being weighed. Following 48 h, blood samples were extracted from the retroorbital venous plexus using light ether anesthesia and the serum was separated by centrifugation in order to measure the glucose level. In order to qualify as diabetic rats for additional testing, only rats with blood glucose levels greater than 200 mg/dl were chosen^12–17^.

### Experimental design

Eight groups, (6–10 diabetic rats, each), were randomly selected. Group I, received only saline and served as the normal control group, whereas Group II (STZ-control), represented the untreated STZ- induced diabetic rats. Group III (STZ-MET) was the standard group receiving metformin (MET; 10 mg/ Kg). The crude extract of *Chaetomium globosum* KM651986 was administered orally to Groups IV (STZ-Extract 100 mg) and V (STZ-Extract 200 mg) at doses of 100 and 200 mg/ Kg, respectively. Compounds **1** and **2** were administered orally to groups VI (STZ-Compound **1**) and VII (STZ-Compound **2**) at doses of 3.5 mg/kg and 2 mg/kg (according to the yield)^12–17^. Compounds 1, 2, the extract, and metformin were administered orally once daily for 14 consecutive days following confirmation of diabetes. Seven animals from each group remained and were used for the collection of blood samples and liver tissue homogenates. All biochemical and statistical analyses were therefore performed using *n* = 7 per group.

### Estimation of biochemical parameters

After 14 consecutive days, blood samples were drawn from rats that had been fasting overnight. Serum levels of insulin, glucose, triglycerides, total cholesterol, and α-amylase were assessed according to^14^. Rats were then sacrificed by being cervically dislocated, liver tissues were collected and homogenized and hepatic reduced glutathione (GSH) was estimated according to^13^.

### Biochemical assays

Insulin: Serum insulin levels were quantified using a rat-specific ELISA kit (Cat. No. E-EL-R2466, Elabscience Biotechnology Inc., Wuhan, China), according to the manufacturer’s instructions. The assay is based on a sandwich ELISA format with a detection range of 0.188–12.0 µIU/mL and a sensitivity of 0.1 µIU/mL. Absorbance was measured at 450 nm using a microplate reader (BioTek Epoch, USA), and sample concentrations were determined via standard curve generation.

HbA1c: Glycated hemoglobin (HbA1c) levels were determined using a commercial colorimetric assay kit (Cat. No. ab65333, Abcam, Cambridge, UK). The assay measures the ratio of HbA1c to total hemoglobin at 575 nm and 540 nm, respectively, and provides a detection range of 0.2–10%. Calibration was done using provided standards, and results were expressed as a percentage of total hemoglobin.

α-Amylase: Hepatic α-amylase activity was assessed using an α-amylase assay kit (Cat. No. MAK009, Sigma-Aldrich, St. Louis, MO, USA). The method is based on the cleavage of a chromogenic substrate and measurement of the resulting product at 405 nm. The assay has a linear range of 0.2–2.0 U/mL. Enzyme activity was normalized to protein content and expressed as U/g of liver tissue.

cAMP: Hepatic cyclic adenosine monophosphate (cAMP) levels were measured using a competitive ELISA kit (Cat. No. ADI-900-066, Enzo Life Sciences, Farmingdale, NY, USA). The assay detects cAMP with high specificity and a sensitivity of 0.1 pmol/mL, with a dynamic range of 0.078–20 pmol/mL. Absorbance was read at 405 nm, and results were calculated based on the standard curve.

### Statistical analysis

All data were expressed as mean ± standard deviation (SD). Statistical comparisons among groups were conducted using one-way ANOVA followed by Tukey’s post hoc test. GraphPad Prism software version 9.0 (GraphPad Software, San Diego, CA, USA) was used to perform all statistical analyses. A p-value of < 0.05 was considered statistically significant.

## Results

### In silico identification of biological targets for compounds 1 and 2

Nine similar compounds with similarity scores ranging from 0.687 to 0.551 were found by using the SwissSimilarity check of compounds **1** and **2** on the ligand Expo database. Thielavins, a substance that was previously identified from *Chaetomium carinthiacum* ATCC 46,463, had the highest similarity hit.

**Fig. 4:**
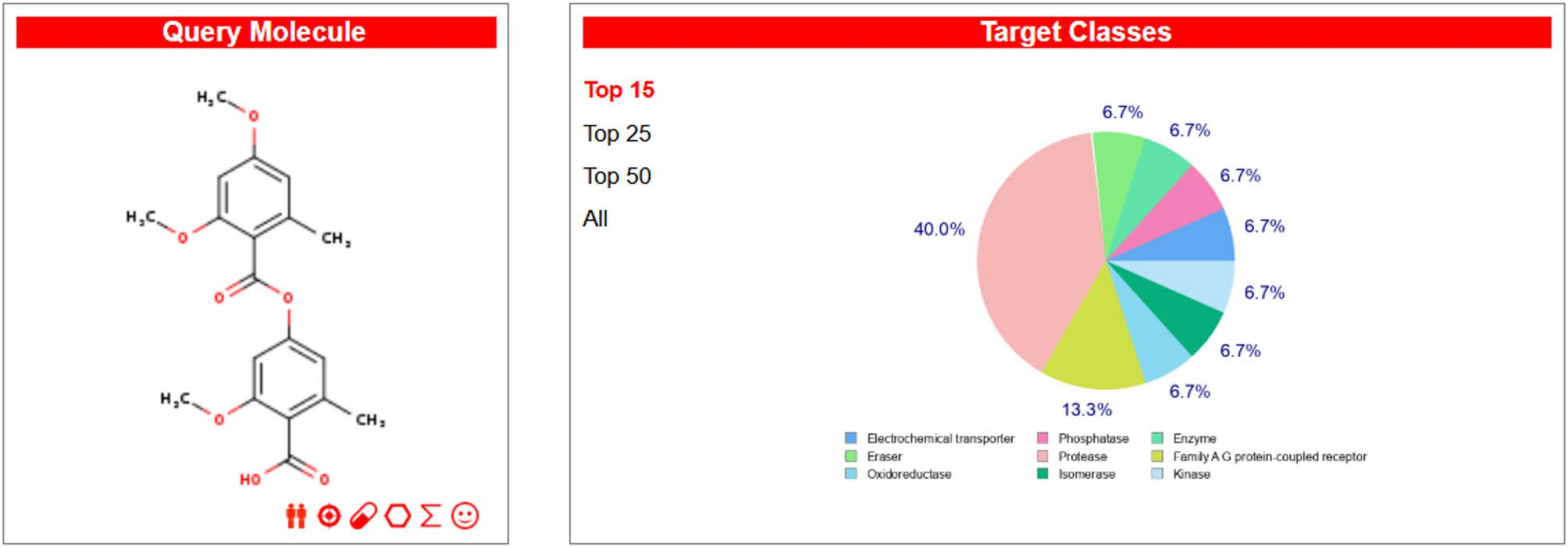
SwissTargetPrediction Results for compound 1.

**Fig. 5:**
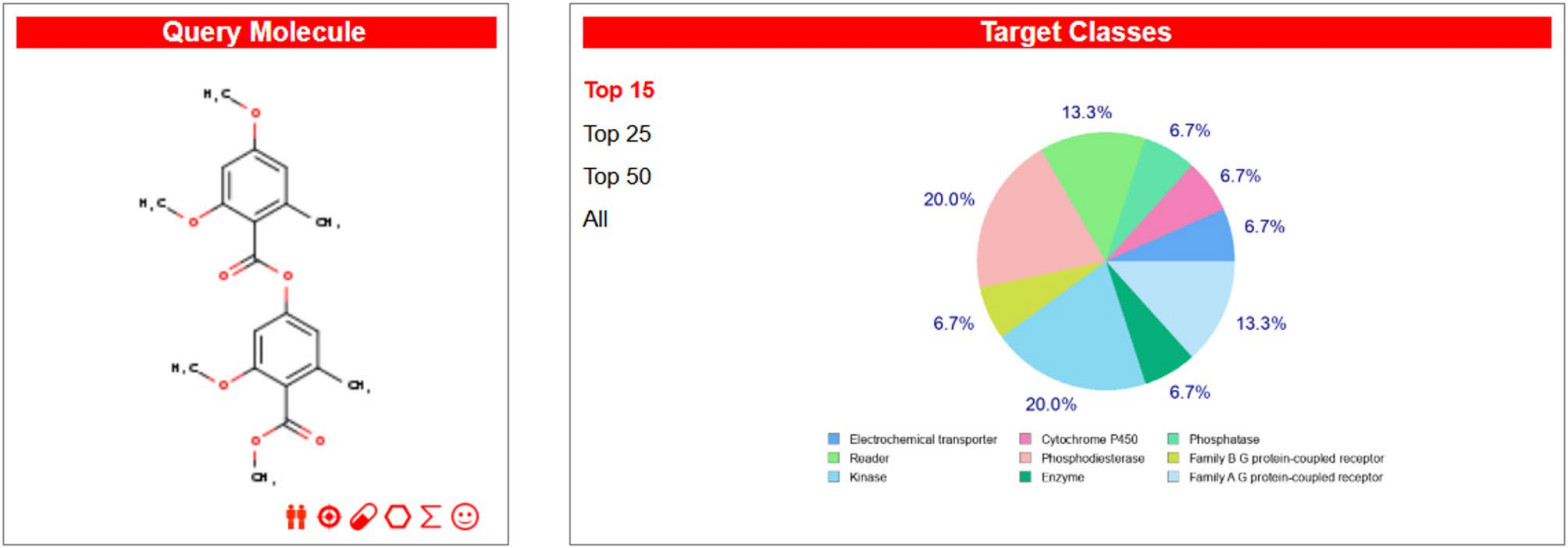
SwissTargetPrediction Results for compound 2.

Compounds **1** and **2** were most likely effective on glucose-6-phosphate translocase (Uniprot id: O43826, ChEMBL ID: CHEMBL3217398), according to a SwissTargetPrediction study, with a probability score of 0.377301276184. The electrochemical transporter target class includes glucose-6-phosphate translocase, which has two known actives in 2D and zero in 3D. In addition to glucose-6-phosphate translocase, SwissTargetPrediction identified 91 other targets, including HMG-CoA reductase, with probability ratings ranging from 1 to 343 for known actives in 3D (Figs. 4 and 5). These results directed us towards anti-diabetic activity due to the essential role of glucose-6-phosphate translocase in glucose metabolism (Tables 2 and 3).

**Table 2:** SwissTargetPrediction results for compound 1.

Target	Common name	Uniprot ID	ChEMBL ID	Target Class	Probability
Glucose-6-phosphate translocase	SLC37A4	O43826	CHEMBL3217398	Electrochemical transporter	0.166154
Protein-tyrosine phosphatase 1B	PTPN1	P18031	CHEMBL335	Phosphatase	0.10934
Prostaglandin E synthase	PTGES	O14684	CHEMBL5658	Enzyme	0.10934
Lysine-specific demethylase 6B	KDM6B	O15054	CHEMBL1938211	Eraser	0.10934
Lysosomal protective protein	CTSA	P10619	CHEMBL6115	Protease	0.10934
Angiotensin-converting enzyme	ACE	P12821	CHEMBL1808	Protease	0.10934
Neprilysin	MME	P08473	CHEMBL1944	Protease	0.10934
Matrix metalloproteinase 2	MMP2	P08253	CHEMBL333	Protease	0.10934
Hydroxycarboxylic acid receptor 2	HCAR2	Q8TDS4	CHEMBL3785	Family A G protein-coupled receptor	0.10934
Caspase-1	CASP1	P29466	CHEMBL4801	Protease	0.10934
Dihydroorotate dehydrogenase	DHODH	Q02127	CHEMBL1966	Oxidoreductase	0.10934
Cyclophilin A	PPIA	P62937	CHEMBL1949	Isomerase	0.10934
c-Jun N-terminal kinase 1	MAPK8	P45983	CHEMBL2276	Kinase	0.10934
Endothelin-converting enzyme 1	ECE1	P42892	CHEMBL4791	Protease	0.10934
C-C chemokine receptor type 1	CCR1	P32246	CHEMBL2413	Family A G protein-coupled receptor	0.10934
Matrix metalloproteinase 9	MMP9	P14780	CHEMBL321	Protease	0.10934
Integrin alpha-V/beta-3	ITGAV ITGB3	P06756 P05106	CHEMBL1907598	Membrane receptor	0.10934
Serine/threonine-protein kinase Aurora-A	AURKA	O14965	CHEMBL4722	Kinase	0.10934
Epoxide hydratase	EPHX2	P34913	CHEMBL2409	Protease	0.10934
Dual specificity mitogen-activated protein kinase kinase 1	MAP2K1	Q02750	CHEMBL3587	Kinase	0.10934
Leukotriene B4 receptor 1	LTB4R	Q15722	CHEMBL3911	Family A G protein-coupled receptor	0.10934
Hexokinase type IV	GCK	P35557	CHEMBL3820	Enzyme	0.10934
Autotaxin	ENPP2	Q13822	CHEMBL3691	Enzyme	0.10934
Matrix metalloproteinase 13	MMP13	P45452	CHEMBL280	Protease	0.10934
Matrix metalloproteinase 3	MMP3	P08254	CHEMBL283	Protease	0.10934
Cytochrome P450 26A1	CYP26A1	O43174	CHEMBL5141	Cytochrome P450	0.10934
Epidermal growth factor receptor erbB1	EGFR	P00533	CHEMBL203	Kinase	0.10934
Serine/threonine-protein kinase Chk1	CHEK1	O14757	CHEMBL4630	Kinase	0.10934
Integrin alpha-4/beta-1	ITGB1 ITGA4	P05556 P13612	CHEMBL1907599	Membrane receptor	0.10934
Protein farnesyltransferase	FNTA FNTB	P49354 P49356	CHEMBL2094108	Enzyme	0.10934
Segment polarity protein dishevelled homolog DVL-1	DVL1	O14640	CHEMBL6027	Unclassified protein	0.10934
MAP kinase p38 alpha	MAPK14	Q16539	CHEMBL260	Kinase	0.10934
Phosphodiesterase 10 A (by homology)	PDE10A	Q9Y233	CHEMBL4409	Phosphodiesterase	0.10934
Mitogen-activated protein kinase kinase kinase 5	MAP3K5	Q99683	CHEMBL5285	Kinase	0.10934
Matrix metalloproteinase 12	MMP12	P39900	CHEMBL4393	Protease	0.10934
Casein kinase II alpha	CSNK2A1	P68400	CHEMBL3629	Kinase	0.10934
Glycogen synthase kinase-3 beta	GSK3B	P49841	CHEMBL262	Kinase	0.10934
Peroxisome proliferator-activated receptor delta	PPARD	Q03181	CHEMBL3979	Nuclear receptor	0.10934
Gonadotropin-releasing hormone receptor	GNRHR	P30968	CHEMBL1855	Family A G protein-coupled receptor	0.10934
Lysine-specific demethylase 2 A	KDM2A	Q9Y2K7	CHEMBL1938210	Eraser	0.10934
Intercellular adhesion molecule (ICAM-1), Integrin alpha-L/beta-2	ITGAL ICAM1 ITGB2	P20701 P05362 P05107	CHEMBL2096661	Membrane receptor	0.10934
Lysine-specific demethylase 5 C	KDM5C	P41229	CHEMBL2163176	Eraser	0.10934
Matrix metalloproteinase 1	MMP1	P03956	CHEMBL332	Protease	0.10934
Integrin alpha-4/beta-7	ITGB7 ITGA4	P26010 P13612	CHEMBL2095184	Membrane receptor	0.10934
ADAMTS5	ADAMTS5	Q9UNA0	CHEMBL2285	Protease	0.10934
Chymase	CMA1	P23946	CHEMBL4068	Protease	0.10934
Prostanoid DP receptor	PTGDR	Q13258	CHEMBL4427	Family A G protein-coupled receptor	0.10934
Nerve growth factor receptor Trk-A	NTRK1	P04629	CHEMBL2815	Kinase	0.10934
DNA polymerase alpha subunit	POLA1	P09884	CHEMBL1828	Transferase	0.10934
DNA polymerase beta	POLB	P06746	CHEMBL2392	Enzyme	0.10934
Inhibitor of nuclear factor kappa B kinase epsilon subunit	IKBKE	Q14164	CHEMBL3529	Kinase	0.10934
Serine/threonine-protein kinase TBK1	TBK1	Q9UHD2	CHEMBL5408	Kinase	0.10934
Dopamine D4 receptor	DRD4	P21917	CHEMBL219	Family A G protein-coupled receptor	0.10934
Dual specificity protein phosphatase 3	DUSP3	P51452	CHEMBL2635	Phosphatase	0.10934
Interleukin-8 receptor B	CXCR2	P25025	CHEMBL2434	Family A G protein-coupled receptor	0.10934
Receptor protein-tyrosine kinase erbB-2	ERBB2	P04626	CHEMBL1824	Kinase	0.10934
Cyclin-dependent kinase 2	CDK2	P24941	CHEMBL301	Kinase	0.10934
Cyclin-dependent kinase 4	CDK4	P11802	CHEMBL331	Kinase	0.10934
Receptor-type tyrosine-protein phosphatase F (LAR)	PTPRF	P10586	CHEMBL3521	Membrane receptor	0.10934
Matrix metalloproteinase 8	MMP8	P22894	CHEMBL4588	Protease	0.10934
Troponin, cardiac muscle	TNNC1 TNNT2 TNNI3	P63316 P45379 P19429	CHEMBL2095202	Unclassified protein	0.10934
Hematopoietic cell protein-tyrosine phosphatase 70Z-PEP	PTPN22	Q9Y2R2	CHEMBL2889	Phosphatase	0.10934
11-beta-hydroxysteroid dehydrogenase 1	HSD11B1	P28845	CHEMBL4235	Enzyme	0.10934
Liver glycogen phosphorylase	PYGL	P06737	CHEMBL2568	Enzyme	0.10934
Glycogen synthase kinase-3 alpha	GSK3A	P49840	CHEMBL2850	Kinase	0.10934
DNA-(apurinic or apyrimidinic site) lyase	APEX1	P27695	CHEMBL5619	Enzyme	0.10934
Cathepsin (V and K)	CTSV	O60911	CHEMBL3272	Protease	0.10934
Coagulation factor V	F5	P12259	CHEMBL3618	Secreted protein	0.10934
Carnitine O-palmitoyltransferase 1, liver isoform	CPT1A	P50416	CHEMBL1293194	Enzyme	0.10934
Carnitine O-palmitoyltransferase 1, muscle isoform	CPT1B	Q92523	CHEMBL2216739	Group translocator	0.10934
Phosphodiesterase 4 A	PDE4A	P27815	CHEMBL254	Phosphodiesterase	0.10934
Tyrosine-protein kinase ABL	ABL1	P00519	CHEMBL1862	Kinase	0.10934
Integrin alpha-V/beta-1	ITGAV ITGB1	P06756 P05556	CHEMBL2111407	Membrane receptor	0.10934
Lysophosphatidic acid receptor Edg-4	LPAR2	Q9HBW0	CHEMBL3724	Family A G protein-coupled receptor	0.10934
Complement factor D	CFD	P00746	CHEMBL2176771	Protease	0.10934
MAP kinase signal-integrating kinase 2	MKNK2	Q9HBH9	CHEMBL4204	Kinase	0.10934
NAD-dependent deacetylase sirtuin 2	SIRT2	Q8IXJ6	CHEMBL4462	Eraser	0.10934
NAD-dependent deacetylase sirtuin 1	SIRT1	Q96EB6	CHEMBL4506	Eraser	0.10934
Transient receptor potential cation channel subfamily M member 8	TRPM8	Q7Z2W7	CHEMBL1075319	Voltage-gated ion channel	0.10934
Endothelin receptor ET-B	EDNRB	P24530	CHEMBL1785	Family A G protein-coupled receptor	0.10934
Phosphodiesterase 4D	PDE4D	Q08499	CHEMBL288	Phosphodiesterase	0.10934
Phosphodiesterase 3	PDE3A	Q14432	CHEMBL241	Phosphodiesterase	0.10934
Phosphodiesterase 7 A	PDE7A	Q13946	CHEMBL3012	Phosphodiesterase	0.10934
Membrane metallo-endopeptidase-like 1	MMEL1	Q495T6	CHEMBL3638356	Hydrolase	0.10934
Carbonic anhydrase VII	CA7	P43166	CHEMBL2326	Lyase	0.10934
Carbonic anhydrase I	CA1	P00915	CHEMBL261	Lyase	0.10934
Carbonic anhydrase XII	CA12	O43570	CHEMBL3242	Lyase	0.10934
Adenosine A2a receptor	ADORA2A	P29274	CHEMBL251	Family A G protein-coupled receptor	0.10934
Cathepsin L	CTSL	P07711	CHEMBL3837	Protease	0.10934
Thromboxane A2 receptor	TBXA2R	P21731	CHEMBL2069	Family A G protein-coupled receptor	0.10934
ADAMTS4	ADAMTS4	O75173	CHEMBL2318	Protease	0.10934
Phosphodiesterase 4B	PDE4B	Q07343	CHEMBL275	Phosphodiesterase	0.10934
6-phosphofructo-2-kinase/fructose-2,6-bisphosphatase 3	PFKFB3	Q16875	CHEMBL2331053	Enzyme	0.10934
Bradykinin B1 receptor	BDKRB1	P46663	CHEMBL4308	Family A G protein-coupled receptor	0.10934
5-lipoxygenase activating protein	ALOX5AP	P20292	CHEMBL4550	Other cytosolic protein	0.10934
Phosphomannomutase 2	PMM2	O15305	CHEMBL1741162	Enzyme	0.10934
Mannose-6-phosphate isomerase	MPI	P34949	CHEMBL2758	Isomerase	0.10934
Phosphoethanolamine/phosphocholine phosphatase	PHOSPHO1	Q8TCT1	CHEMBL6113	Enzyme	0.10934
Integrin alpha-4	ITGA4	P13612	CHEMBL278	Membrane receptor	0.10934
G-protein coupled receptor kinase 2	GRK2	P25098	CHEMBL4079	Kinase	0.10934

**Table 3:** SwissTargetPrediction results for compound 2.

Target	Common name	Uniprot ID	ChEMBL ID	Target Class	Probability
Glucose-6-phosphate translocase	SLC37A4	O43826	CHEMBL3217398	Electrochemical transporter	0.161276819
Cytochrome P450 19A1	CYP19A1	P11511	CHEMBL1978	Cytochrome P450	0.121287003
Protein-tyrosine phosphatase 1B	PTPN1	P18031	CHEMBL335	Phosphatase	0.113285953
Bromodomain-containing protein 9	BRD9	Q9H8M2	CHEMBL3108640	Reader	0.113285953
Phosphodiesterase 3	PDE3A	Q14432	CHEMBL241	Phosphodiesterase	0.113285953
Phosphodiesterase 3B	PDE3B	Q13370	CHEMBL290	Phosphodiesterase	0.113285953
Phosphodiesterase 10 A	PDE10A	Q9Y233	CHEMBL4409	Phosphodiesterase	0.113285953
Corticotropin releasing factor receptor 1	CRHR1	P34998	CHEMBL1800	Family B G protein-coupled receptor	0.113285953
Leucine-rich repeat serine/threonine-protein kinase 2	LRRK2	Q5S007	CHEMBL1075104	Kinase	0.113285953
Vascular endothelial growth factor receptor 1	FLT1	P17948	CHEMBL1868	Kinase	0.113285953
Cell division protein kinase 8	CDK8	P49336	CHEMBL5719	Kinase	0.113285953
Prostaglandin E synthase	PTGES	O14684	CHEMBL5658	Enzyme	0.113285953
Bromodomain-containing protein 2	BRD2	P25440	CHEMBL1293289	Reader	0.113285953
Adenosine A2a receptor	ADORA2A	P29274	CHEMBL251	Family A G protein-coupled receptor	0.113285953
Adenosine A2b receptor	ADORA2B	P29275	CHEMBL255	Family A G protein-coupled receptor	0.113285953
Phosphodiesterase 4B	PDE4B	Q07343	CHEMBL275	Phosphodiesterase	0.113285953
Phosphodiesterase 7 A	PDE7A	Q13946	CHEMBL3012	Phosphodiesterase	0.113285953
Translocator protein (by homology)	TSPO	P30536	CHEMBL5742	Membrane receptor	0.113285953
CDC7/DBF4 (Cell division cycle 7-related protein kinase/Activator of S phase kinase)	DBF4 CDC7	Q9UBU7 O00311	CHEMBL2111377	Kinase	0.113285953
MAP kinase p38 alpha	MAPK14	Q16539	CHEMBL260	Kinase	0.113285953
Voltage-gated calcium channel alpha2/delta subunit 1	CACNA2D1	P54289	CHEMBL1919	Calcium channel auxiliary subunit alpha2delta family	0.113285953
Potassium channel subfamily K member 3	KCNK3	O14649	CHEMBL2321613	Voltage-gated ion channel	0.113285953
Potassium channel subfamily K member 9	KCNK9	Q9NPC2	CHEMBL2321614	Voltage-gated ion channel	0.113285953
Metabotropic glutamate receptor 1	GRM1	Q13255	CHEMBL3772	Family C G protein-coupled receptor	0.113285953
Voltage-gated calcium channel alpha2/delta subunit 2	CACNA2D2	Q9NY47	CHEMBL3896	Calcium channel auxiliary subunit alpha2delta family	0.113285953
Glutaminyl-peptide cyclotransferase	QPCT	Q16769	CHEMBL4508	Enzyme	0.113285953
Serine/threonine-protein kinase B-raf	BRAF	P15056	CHEMBL5145	Kinase	0.113285953
Serine/threonine-protein kinase TNNI3K	TNNI3K	Q59H18	CHEMBL5260	Kinase	0.113285953
Phosphoglycerate kinase 1	PGK1	P00558	CHEMBL2886	Enzyme	0.113285953
Epidermal growth factor receptor erbB1	EGFR	P00533	CHEMBL203	Kinase	0.113285953
Orexin receptor 2	HCRTR2	O43614	CHEMBL4792	Family A G protein-coupled receptor	0.113285953
Orexin receptor 1	HCRTR1	O43613	CHEMBL5113	Family A G protein-coupled receptor	0.113285953
Serine/threonine protein phosphatase PP1-alpha catalytic subunit	PPP1CA	P62136	CHEMBL2164	Phosphatase	0.113285953
Hexokinase type IV	GCK	P35557	CHEMBL3820	Enzyme	0.113285953
N(G), N(G)-dimethylarginine dimethylaminohydrolase 1	DDAH1	O94760	CHEMBL6036	Enzyme	0.113285953
Monoamine oxidase B	MAOB	P27338	CHEMBL2039	Oxidoreductase	0.113285953
Gamma-secretase	PSEN2 PSENEN NCSTN APH1A PSEN1 APH1B	P49810 Q9NZ42 Q92542 Q96BI3 P49768 Q8WW43	CHEMBL2094135	Protease	0.113285953
c-Jun N-terminal kinase 1	MAPK8	P45983	CHEMBL2276	Kinase	0.113285953
Vanilloid receptor	TRPV1	Q8NER1	CHEMBL4794	Voltage-gated ion channel	0.113285953
Glycogen synthase kinase-3 beta	GSK3B	P49841	CHEMBL262	Kinase	0.113285953
Estradiol 17-beta-dehydrogenase 2	HSD17B2	P37059	CHEMBL2789	Enzyme	0.113285953
Aldehyde dehydrogenase 1A1	ALDH1A1	P00352	CHEMBL3577	Enzyme	0.113285953
PI3-kinase p110-alpha subunit	PIK3CA	P42336	CHEMBL4005	Enzyme	0.113285953
Neuropeptide Y receptor type 5	NPY5R	Q15761	CHEMBL4561	Family A G protein-coupled receptor	0.113285953
G-protein coupled receptor 55	GPR55	Q9Y2T6	CHEMBL1075322	Family A G protein-coupled receptor	0.113285953
c-Jun N-terminal kinase 3	MAPK10	P53779	CHEMBL2637	Kinase	0.113285953
P2X purinoceptor 7	P2RX7	Q99572	CHEMBL4805	Ligand-gated ion channel	0.113285953
Proto-oncogene tyrosine-protein kinase MER	MERTK	Q12866	CHEMBL5331	Kinase	0.113285953
Tubulin beta-1 chain	TUBB1	Q9H4B7	CHEMBL1915	Structural protein	0.113285953
GABA-A receptor; alpha-2/beta-3/gamma-2	GABRA2 GABRB3 GABRG2	P47869 P28472 P18507	CHEMBL2094130	Ligand-gated ion channel	0.113285953
Anandamide amidohydrolase	FAAH	O00519	CHEMBL2243	Enzyme	0.113285953
6-phosphofructo-2-kinase/fructose-2,6-bisphosphatase 3	PFKFB3	Q16875	CHEMBL2331053	Enzyme	0.113285953
Neuronal acetylcholine receptor protein alpha-7 subunit	CHRNA7	P36544	CHEMBL2492	Ligand-gated ion channel	0.113285953
TGF-beta receptor type I	TGFBR1	P36897	CHEMBL4439	Kinase	0.113285953
Nicotinamide phosphoribosyltransferase	NAMPT	P43490	CHEMBL1744525	Enzyme	0.113285953
Melatonin receptor 1 A	MTNR1A	P48039	CHEMBL1945	Family A G protein-coupled receptor	0.113285953
Melatonin receptor 1B	MTNR1B	P49286	CHEMBL1946	Family A G protein-coupled receptor	0.113285953
Sodium channel protein type V alpha subunit	SCN5A	Q14524	CHEMBL1980	Voltage-gated ion channel	0.113285953
Serotonin 2a (5-HT2a) receptor	HTR2A	P28223	CHEMBL224	Family A G protein-coupled receptor	0.113285953
Serotonin 6 (5-HT6) receptor	HTR6	P50406	CHEMBL3371	Family A G protein-coupled receptor	0.113285953
Dual specificity mitogen-activated protein kinase kinase 1	MAP2K1	Q02750	CHEMBL3587	Kinase	0.113285953
Sodium channel protein type II alpha subunit	SCN2A	Q99250	CHEMBL4187	Voltage-gated ion channel	0.113285953
Sodium channel protein type IX alpha subunit	SCN9A	Q15858	CHEMBL4296	Voltage-gated ion channel	0.113285953
Sodium channel protein type III alpha subunit	SCN3A	Q9NY46	CHEMBL5163	Voltage-gated ion channel	0.113285953
Sodium channel protein type VIII alpha subunit	SCN8A	Q9UQD0	CHEMBL5202	Voltage-gated ion channel	0.113285953
Epoxide hydratase	EPHX2	P34913	CHEMBL2409	Protease	0.113285953
Interleukin-8 receptor B	CXCR2	P25025	CHEMBL2434	Family A G protein-coupled receptor	0.113285953
PI3-kinase p110-delta subunit	PIK3CD	O00329	CHEMBL3130	Enzyme	0.113285953
PI3-kinase p110-beta subunit	PIK3CB	P42338	CHEMBL3145	Enzyme	0.113285953
PI3-kinase p110-gamma subunit	PIK3CG	P48736	CHEMBL3267	Enzyme	0.113285953
DNA-directed RNA polymerase I subunit RPA1	POLR1A	O95602	CHEMBL3286067	Enzyme	0.113285953
c-Jun N-terminal kinase 2	MAPK9	P45984	CHEMBL4179	Kinase	0.113285953
Receptor protein-tyrosine kinase erbB-2	ERBB2	P04626	CHEMBL1824	Kinase	0.113285953
Tyrosine-protein kinase LCK	LCK	P06239	CHEMBL258	Kinase	0.113285953
Phosphodiesterase 4D	PDE4D	Q08499	CHEMBL288	Phosphodiesterase	0.113285953
MAP kinase p38 beta	MAPK11	Q15759	CHEMBL3961	Kinase	0.113285953
Voltage-gated potassium channel subunit Kv1.5	KCNA5	P22460	CHEMBL4306	Voltage-gated ion channel	0.113285953
Proteasome Macropain subunit MB1	PSMB5	P28074	CHEMBL4662	Protease	0.113285953
Probable protein-cysteine N-palmitoyltransferase porcupine (by homology)	PORCN	Q9H237	CHEMBL1255163	Enzyme	0.113285953
MAP kinase-activated protein kinase 2	MAPKAPK2	P49137	CHEMBL2208	Kinase	0.113285953
Cyclin-dependent kinase 2	CDK2	P24941	CHEMBL301	Kinase	0.113285953
Rho-associated protein kinase 1	ROCK1	Q13464	CHEMBL3231	Kinase	0.113285953
Cyclin-dependent kinase 4	CDK4	P11802	CHEMBL331	Kinase	0.113285953
Cytochrome P450 17A1	CYP17A1	P05093	CHEMBL3522	Cytochrome P450	0.113285953
cAMP-dependent protein kinase alpha-catalytic subunit	PRKACA	P17612	CHEMBL4101	Kinase	0.113285953
CDC7/DBF4 (Cell division cycle 7-related protein kinase/Activator of S phase kinase)	CDC7	O00311	CHEMBL5443	Kinase	0.113285953
Mineralocorticoid receptor	NR3C2	P08235	CHEMBL1994	Nuclear receptor	0.113285953
Phosphodiesterase 4 A	PDE4A	P27815	CHEMBL254	Phosphodiesterase	0.113285953
Phosphodiesterase 4 C	PDE4C	Q08493	CHEMBL291	Phosphodiesterase	0.113285953
Intercellular adhesion molecule-1	ICAM1	P05362	CHEMBL3070	Adhesion	0.113285953
Phosphodiesterase 9 A	PDE9A	O76083	CHEMBL3535	Phosphodiesterase	0.113285953
Vascular cell adhesion protein 1	VCAM1	P19320	CHEMBL3735	Adhesion	0.113285953
Selectin E	SELE	P16581	CHEMBL3890	Adhesion	0.113285953
11-beta-hydroxysteroid dehydrogenase 1	HSD11B1	P28845	CHEMBL4235	Enzyme	0.113285953
G-protein coupled bile acid receptor 1	GPBAR1	Q8TDU6	CHEMBL5409	Family A G protein-coupled receptor	0.113285953
Neuropeptides B/W receptor type 1	NPBWR1	P48145	CHEMBL1293293	Family A G protein-coupled receptor	0.113285953
FK506-binding protein 1 A	FKBP1A	P62942	CHEMBL1902	Isomerase	0.113285953
Cyclin-dependent kinase 1	CDK1	P06493	CHEMBL308	Kinase	0.113285953
Smoothened homolog	SMO	Q99835	CHEMBL5971	Frizzled family G protein-coupled receptor	0.113285953
Cytochrome P450 11B1	CYP11B1	P15538	CHEMBL1908	Cytochrome P450	0.113285953

### Anti-diabetic properties

#### Effects of the crude extract of *Chaetomium globosum* KM651986 and compounds 1 and 2 on serum glucose and insulin levels in STZ-induced diabetic rats

**Fig. 6:**
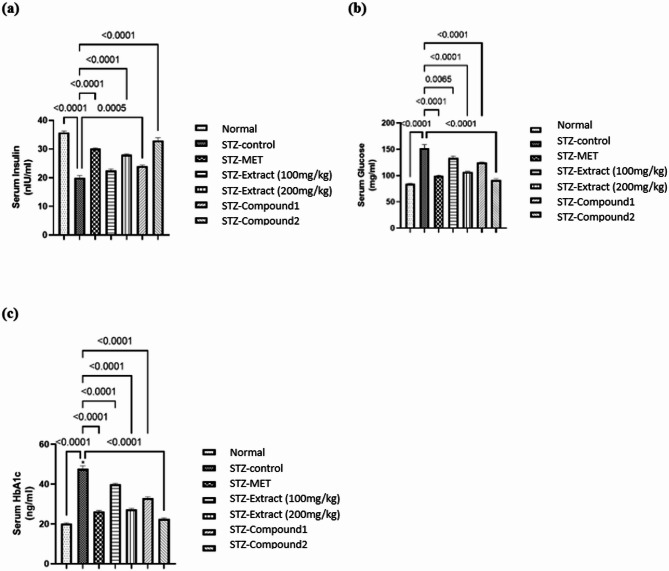
Effects of the crude extract of *Chaetomium globosum* KM651986 and compounds 1 and 2 on (**a**) serum glucose, (**b**) serum insulin and (**c**) serum HbA1c in STZ-induced diabetic rats. Data are expressed as mean ± SEM (*n* = 7/group). Statistical analysis was performed using one-way ANOVA followed by Tukey’s post hoc test. Exact* p*-values are shown on the graph where significant differences were detected.

STZ intraperitoneal injection led to increase in serum glucose levels of rats by 1.8 times their normal levels. However, treatment of STZ- diabetic rats with the crude extract of *C. globosum* (100 and 200 mg/kg) decreased serum glucose levels by approximately 12% and 29%, respectively. Whereas the serum glucose level of rats treated with **1** and **2** significantly decreased by about 18% and 40%, respectively (Fig. 6a).

STZ injection also reduced serum insulin levels markedly by 45%, compared to normal. Administration of the crude extract of *C. globosum* (100 and 200 mg/kg) to STZ- diabetic rats increased serum insulin levels by about 40%, while compounds **1** and **2** increased significantly insulin levels by nearly 20% and 65%, respectively as compared to the diabetic rats (Fig. 6b).

#### Effects of the crude extract of *Chaetomium globosum* KM651986 and compounds 1 and 2 on serum HbA1c level in STZ-induced diabetic rats

Serum HbA1C was increased in diabetic rats after STZ injection by approximately 2.3 times normal rats. The crude extract of *C. globosum* (100 and 200 mg/kg) reduced serum HbA1C by about 16% and 43%, however, treatment with **1** and **2** exhibited a significant decrease in the HbA1c level by about 31% and 53%, respectively as compared to the diabetic rats (Fig. 6c).

#### Effects of the crude extract of *Chaetomium globosum* KM651986 and compounds 1 and 2 on hepatic G6PD content in STZ-induced diabetic rats

**Fig. 7:**
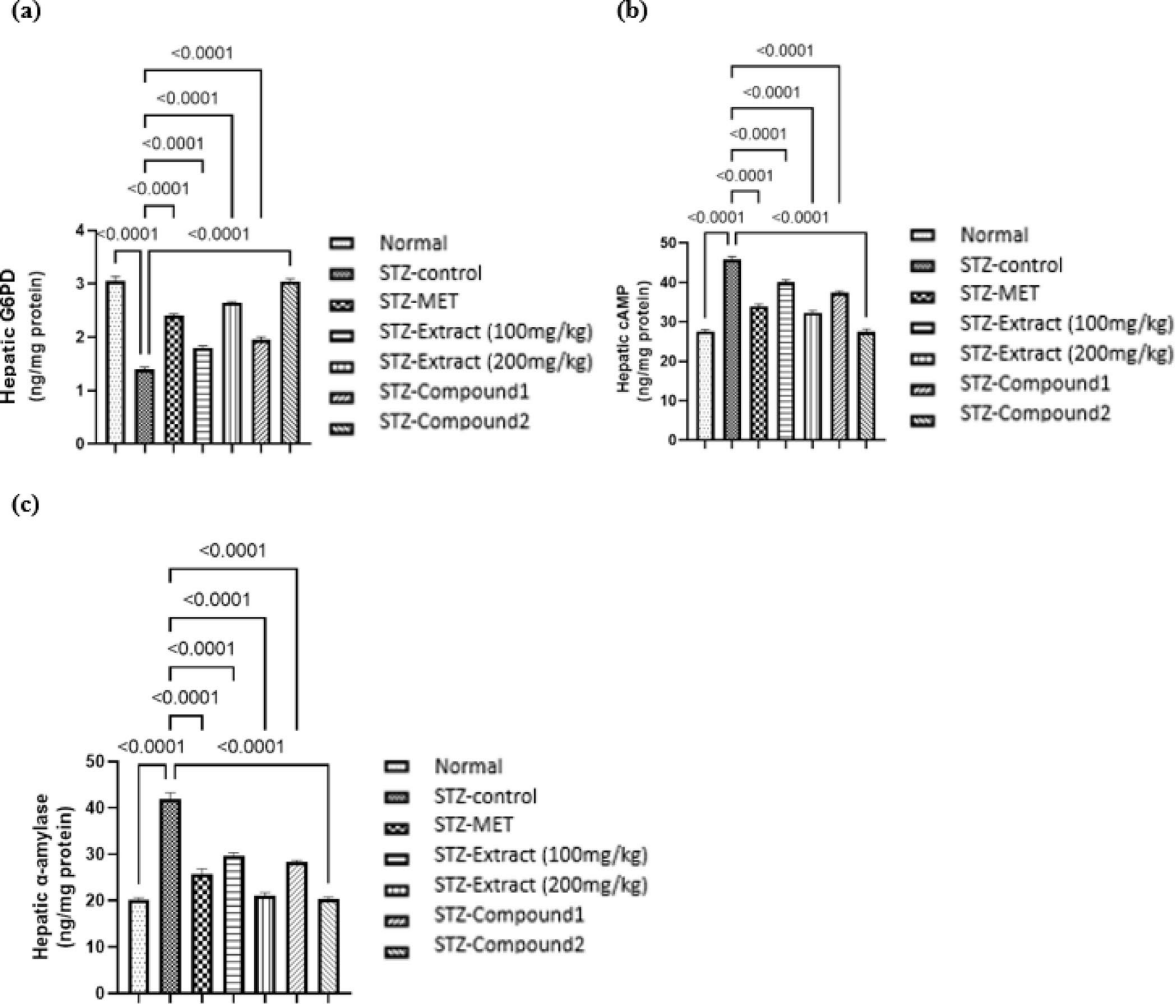
Effects of the crude extract of *Chaetomium globosum* KM651986 and compounds 1 and 2 on (**a**) hepatic G6PD, (**b**) hepatic cAMP, and (**c**) hepatic α-amylase in STZ-induced diabetic rats. Data are expressed as mean ± SEM (*n* = 7/group). Statistical analysis was performed using one-way ANOVA followed by Tukey’s post hoc test. Exact *p*-values are shown on the graph where significant differences were detected.

Figure 7a showed that induction of diabetes was accompanied with a decline in the hepatic G6PD content by about 54% as compared to normal control. Treatment of STZ- diabetic rats with the crude extract of *C. globosum* (100 and 200 mg/kg) showed an elevated hepatic G6PD by about 29% and 89%, whereas treatment with **1** and **2** exhibited a significant decrease in the hepatic G6PD level by about 39% and 117%, respectively as compared to the diabetic rats which are comparable to that of metformin.

#### Effects of the crude extract of *Chaetomium globosum* KM651986 and compounds 1 and 2 on hepatic cAMP level in STZ-induced diabetic rats

Figure 7b presented that diabetes was accompanied with an elevation in the hepatic cAMP level by about 1.6 folds of the normal levels. Treatment of STZ- diabetic rats with the crude extract of *C. globosum* (100 and 200 mg/kg) showed a decrease in the hepatic cAMP by about 13% and 29%, respectively. Similarly, treatment with **1** exhibited a significant decrease in the hepatic cAMP level by about 18% as compared to the diabetic rats. These results were comparable to that of metformin. Correspondingly, **2** succeeded to normalize the hepatic cAMP level.

#### Effects of the crude extract of *Chaetomium globosum* KM651986 and compounds 1 and 2 on hepatic α-amylase content in STZ-induced diabetic rats

Diabetes was also accompanied with an increase in the hepatic α-amylase level by 2 folds of the normal value. On the other hand, treatment with the crude extract of *C. globosum* (100 and 200 mg/kg) showed a decline in the α-amylase level by about 29% and 50%, respectively. Likewise, treatment with **1** and **2** exhibited a significant decrease in the hepatic cAMP level by about 32% and 52%, respectively as compared to the diabetic rats (Fig. 7c). These results were comparable to that of metformin.

## Discussion

Fungi represent an underexplored yet promising reservoir of bioactive metabolites for diabetes management. The Ascomycota fungi, mainly *Chaetomium*, *Cordyceps* and *Aspergillus* exhibit remarkable potential as sources of multi-target antidiabetic agents. These fungi produce bioactive metabolites such as cordycepin, chaetoglobosins, and asterriquinones, which modulate key pathways in type 2 diabetes, including α-amylase and α-glucosidase inhibition, AMP-activated protein kinase (AMPK)-mediated insulin sensitization, peroxisome proliferator-activated receptor gamma (PPAR-γ) activation and protein tyrosine phosphatase 1B (PTP1B) inhibition. Compared to synthetic drugs, fungal anti-diabetics possess unique chemical structures and often demonstrate lower toxicity and broader mechanisms of action due to their ability to lower blood glucose and lipid levels while reserving renal and hepatic functions. However, metabolic variability among strains, poor bioavailability, and limited clinical studies represent major challenges for their use as anti-diabetic agents^19,20^.

*Chaetomium*, particularly *C. globosum*, produce a wide array of over 200 structurally diverse secondary metabolites with significant therapeutic potential. These fungi have been systematically studied, demonstrating promising antimicrobial, antifungal, antimalarial and antiviral effects at low doses. The chemical profile of *Chaetomium* includes several important secondary metabolite classes, including chaetoglobosins, azaphilones, anthraquinones (e.g. emodin and chrysophanol), diketopiperazines, terpenoids and steroids. These metabolites exhibit a broad spectrum of biological activities notably anticancer properties with polysaccharides from *C. globosum* CGMCC 6882 showing significant activity against human lung cancer A549 cells. A study performed by Goda et al. (2023) revealed that Egyptian *C. globosum* isolates, particularly from extreme (e.g., Upper Egypt and Sinai), exhibited potential antimicrobial activities against multi-drug resistant Gram-positive and Gram-negative bacteria which could be attributed to the presence of active antimicrobial compounds e.g. chaetoviridin A, chaetomugilide A, chaetoglobinol A and prochaetoviridin A, tentatively identified by LC-MS/MS. The structural diversity and biological potency of *Chaetomium* compounds highlight their potential for the development of novel therapeutic agents attracting research interest for pharmaceutical applications^21^. Furthermore, Qi et al. (2020) performed a bioactive tracking strategy combining liquid-liquid extraction and high-speed counter-current chromatography (HSCCC) to isolate natural products from *C. globosum* TY1, of *Ginkgo biloba*. The study yielded the novel polyketide chaetoglobol acid, with a rare C11 skeleton, along with other metabolites including chlorinated azaphilones and cytochalasans. Chaetoglobol acid exhibited potent dual inhibition of α-glucosidase (IC50 = 3.04 µM, 18× more active than acarbose) and α-amylase (IC50 = 22.18 µM) in in vitro bioassays, suggesting its potential as an antidiabetic lead for type 2 diabetes management^22^.

Knowledge-based tools are established to computationally determine targets for molecules, either secondary targets for known molecules or new targets for uncharacterized molecules. One of these online web tools is SwissSimilarity, which provides a valuable resource for drug discovery and design as it uses 2D and 3D molecular similarity-based methods to identify compounds with potential biological activity similar to a given query molecule. SwissSimilarity also screens different libraries for a vast collection of compounds, which could be divided to four main groups: (1) “Drugs” referring to approved, experimental and withdrawn drugs, in addition to potential drugs that have advanced to clinical trials, (2) “Bioactives”, such as the Ligand Expo and the Chemical Entities of Biological Interest (ChEBI) collections, as well as ChEMBL29 database compounds and GPCR–Ligand Association (GLASS) database, and the Human Metabolome Database; (3) “Commercials” from various ZINC subsets and vendor catalogs; (4) “Synthesizable” that can be readily synthesized and provided by a shortlist of vendors, especially on top of ZINC (drug-like, lead-like, and fragments), molecules in the following commercially available chemical libraries could be screened: Enamine, ChemBridge, Maybridge, Asinex, AsisChem, Otava, SPECS, TimTec, Vitas, Life Chemicals, ChemDiv, and Innovapharm. Furthermore, searches can be conducted in targeted and focused libraries that contain bioactive molecules which target GPCRs, kinases, and proteases. Thus, accelerating the identification of promising candidates for drug discovery by identifying hits for a certain target, improving the characteristics of known lead compounds, finding new therapeutic uses for existing drugs, as well as investigating structure-activity relationships and exploring chemical space^7^.

Identifying the targeted proteins is crucial to discover, develop or repurpose bioactive molecules. Bio-/chemo-informatics approaches could effectively inspect the most probable targets of small molecules. These targeted prediction techniques (referred to as target fishing) can be categorized into two main groups: structure-based and ligand- based prediction methods^7^. Ligand-based prediction methods have demonstrated remarkable performance and speed in accurately identifying the protein targets of compounds. The intuitive “molecular similarity hypothesis,” which suggests that similar chemicals target common proteins, was validated through the assessment of similarity between compounds using several methods^7,8^. SwissTargetPrediction is also an important web- based tool which utilizes a combination of 2D and 3D similarity metrics to precisely to perform ligand-based target prediction for bioactive small molecules. It supports predictions across multiple species, including humans, and ranks targets by probability scores. Swiss Target Prediction can be accessed at http://www.swisstargetprediction.ch freely without requiring a login^7^. It has wide applications in drug discovery for identifying target proteins, understanding off-target effects, and exploring the mechanism of action for bioactive molecules. SwissTargetPrediction quantifies similarity in a 2-fold approach: a 2D Similarity, which is based on the Tanimoto index, using path-based binary fingerprints (FP2) to compare molecular structures^18^ and a 3D Similarity which uses Manhattan distance on Electroshape 5D (ES5D) vectors incorporating atomic Cartesian coordinates, partial charges, and lipophilic contributions across 20 previously generated molecular conformations^22,23^. For both 2D and 3D similarity measures, the concept is that two molecules that are similar are represented by equivalent vectors that have a quantitative similarity near 1. SwissTargetPrediction uses a Combined-Score to predict common protein targets among molecules. A score above 0.5 indicates a shared target, allowing for high performance in predicting macromolecular targets in various test sets due to complementary 2D and 3D descriptions^23,24^. In the current study, a newly identified strain of *Chaetomium globosum* (KM651986) was investigated resulting in the isolation of two unreported lecanoric acid derivatives **1** and **2**, for the first time. SwissSimilarity was used to identify compounds similar in structure to **1** and **2**, yielding nine compounds with thielavins, previously isolated from *Chaetomium carinthiacum* ATCC 4646, having the highest similarity hit. SwissTargetPrediction predicted the possible biological targets for **1** and **2**, chiefly glucose-6-phosphate translocase, which is important in maintaining blood glucose levels, its dysregulation contributes to hyperglycemia and insulin resistance in type 2 diabetes^25,26^. Consequently, this study investigated the effects of the crude extract of *C. globosum* (KM651986) and compounds **1** and **2** on various biochemical parameters related to diabetes. First, the safety of the crude methanol extract of *Chaetomium globosum* (KM651986) was established through a preliminary acute toxicity study following OECD guidelines. A single oral dose of 2000 mg/kg resulted in no observable signs of toxicity or mortality over a 14-day period, suggesting that the extract is well-tolerated at this dose. This finding substantiates the use of the extract in subsequent pharmacological testing and addresses concerns regarding its safety profile. Nevertheless, we acknowledge the importance of chronic toxicity evaluations and plan to include detailed sub-chronic and histopathological assessments in future studies.

Streptozotocin (STZ)-induced diabetic rat model was employed in the study for several reasons STZ is a potent chemotoxic agent that selectively induces β-cell destruction in the pancreas by alkylating DNA, leading to impaired insulin secretion and the development of chronic hyperglycemia^27^. (STZ)-induced diabetic rat model closely resembles the pathophysiology of type 1 diabetes, characterized by a deficiency in insulin production due to autoimmune destruction of pancreatic β-cells. The STZ model is widely utilized for screening potential hypoglycemic agents, as well as for evaluating compounds with β-cell-protective or regenerative properties^24^. Its established reproducibility, well-documented alterations in glucose metabolism, and alignment with the mechanisms underlying insulin deficiency make it an ideal system for the initial pharmacological assessment of novel anti-diabetic candidates^28^. The results indicate significant impacts on serum glucose, insulin, HbA1c levels, hepatic G6PD content, cAMP levels, and hepatic α-amylase content. Allied with several previous studies, diabetic rats exhibited an increase in serum glucose levels, following STZ injection, indicative of diabetes^12–17^.

Treatment with the crude extract, as well as compounds **1** and **2** resulted in a notable reduction in serum glucose levels. Particularly, compound **2** demonstrated a substantial decrease of 40%, suggesting a potent anti-hyperglycemic effect. Additionally, diabetic rats showed a reduction in serum insulin levels, and the administration of the crude extract and fractions led to an increase in insulin levels. Notably, compound **2** exhibited a remarkable 65% increase compared to diabetic rats, highlighting its potential in improving insulin secretion. In this study, rats induced with STZ exhibited reduced blood insulin levels compared to diabetic control rats. However, the administration of compound **2** significantly enhanced insulin levels in STZ-induced rats. Rats treated with metformin also demonstrated insulin levels comparable to those treated with compound **2**. This insulin-mimicking effect is further heightened by its ability to regenerate insulin-producing cells.

The notable increase in blood insulin levels in STZ-treated rats suggests that *C. globosum* (KM651986)’s hypoglycemic impact is attributed to the stimulation and potentiation of insulin release from the remaining cells of the islets of Langerhans. This study also evaluated the impact on long-term glucose control by assessing serum HbA1c levels. STZ-induced diabetic rats demonstrated an elevation in HbA1c levels, and treatment with the crude extract and isolated compounds resulted in significant reductions. Compound **2** exhibited the most pronounced effect, reducing HbA1c levels by 53% as compared to STZ- diabetic rats.

Furthermore, the study explored hepatic parameters associated with glucose metabolism. Diabetes induction led to a decline in hepatic G6PD content, an enzyme crucial for glucose metabolism. G6PD is a rate limiting enzyme of the pentose phosphate pathway and produces the majority of nicotinamide adenine dinucleotide phosphate (NADPH) in the cell, which is the most abundant reducing coenzyme in the cells. In DM, prior research had shown that G6PD activity was inhibited, and the content of NADPH was decreased in the livers of rats with Type 1 DM induced by STZ treatment^25^. Similar observations have been noted in the livers of individuals with chronic DM^29^. In an animal study inducing mild, moderate, and severe hyperglycemia through STZ and nicotinamide treatment, it was observed that hepatocyte G6PD activity levels in mild hyperglycemia remained comparable to normal values. However, in cases of moderate and severe hyperglycemia, these levels were significantly reduced^30^. On the other hand, treatment with the crude extract and isolated compounds **1** and **2** resulted in a substantial increase in hepatic G6PD levels. Compound **2** displayed the most significant elevation, indicating its potential in improving glucose metabolism at the hepatic level.

Hepatic gluconeogenesis is physiologically initiated by glucagon, which activates adenylyl cyclase (AC) to increase the cAMP level via its receptor on the hepatocyte plasma membrane. cAMP stimulates PKA to phosphorylate cyclic AMP response element binding (CREB), a transcriptional factor that regulates gluconeogenetic genes, and thus increases gluconeogenesis flux^31^. In terms of hepatic cAMP levels, diabetes was associated with an elevation, and treatment with the crude extract and fractions resulted in a decrease. Compound **2** successfully normalized cAMP levels, while both **1** and **2** demonstrated comparable results to metformin treatment. Moreover, diabetes led to an increase in hepatic α-amylase levels, and treatment with the crude extract and fractions resulted in a decline. Compound **2** showed the most significant decrease of 52%, suggesting its effectiveness in mitigating the elevation of hepatic α-amylase associated with diabetes.

## Conclusion

This study provides comprehensive evidence supporting the anti-diabetic effects of the crude extract of *C. globosum* and its compounds **1** and **2** in STZ-induced diabetic rats. These effects encompass improvements in serum glucose, insulin, and HbA1c levels, as well as enhancements in hepatic G6PD content, normalization of cAMP levels, and reduction in hepatic α-amylase content. Compound **2** emerges as particularly promising across multiple parameters, warranting further investigation into its potential therapeutic mechanisms of action.

## Electronic supplementary material

Below is the link to the electronic supplementary material.

Supplementary Material 1

## Data Availability

Data is provided within supplementary information file.
