# Supplementary material for: Two unreported lecanoric acid derivatives isolated from Chaetomium globosum KM651986 and their anti-diabetic effect
Source: Sci Rep. 2025 Jul 2;15:23554. doi: 10.1038/s41598-025-05875-4 (PMC12223274; doi:10.1038/s41598-025-05875-4)
Supplement: Supplementary file 1 — Supplementary Material 1 [file 41598_2025_5875_MOESM1_ESM.pdf]

Supporting information

Two unreported lecanoric acid derivatives isolated from *Chaetomium globosum* KM651986 and their antidiabetic effect

Authors

**S1**	^{1}H NMR spectrum of compound **1** in $CDCl_3$
**S2**	^{13}C NMR spectrum of compound **1** in $CDCl_3$
**S3**	HMQC spectrum of compound **1** in $CDCl_3$
**S4**	HMBC spectrum of compound **1** in $CDCl_3$
**S5**	^{1}H NMR spectrum of compound **2** in $CDCl_3$
**S6**	^{13}C NMR spectrum of compound **2** in $CDCl_3$
**S7**	HMQC spectrum of compound **2** in $CDCl_3$
**S8**	HMBC spectrum of compound **2** in $CDCl_3$
**S9**	HPLC chromatogram for compound **1** purification
**S10**	HPLC chromatogram for compound **2** purification
**S11**	Virtual Similarity Screening and Target Prediction Methodology for compounds 1 and 2

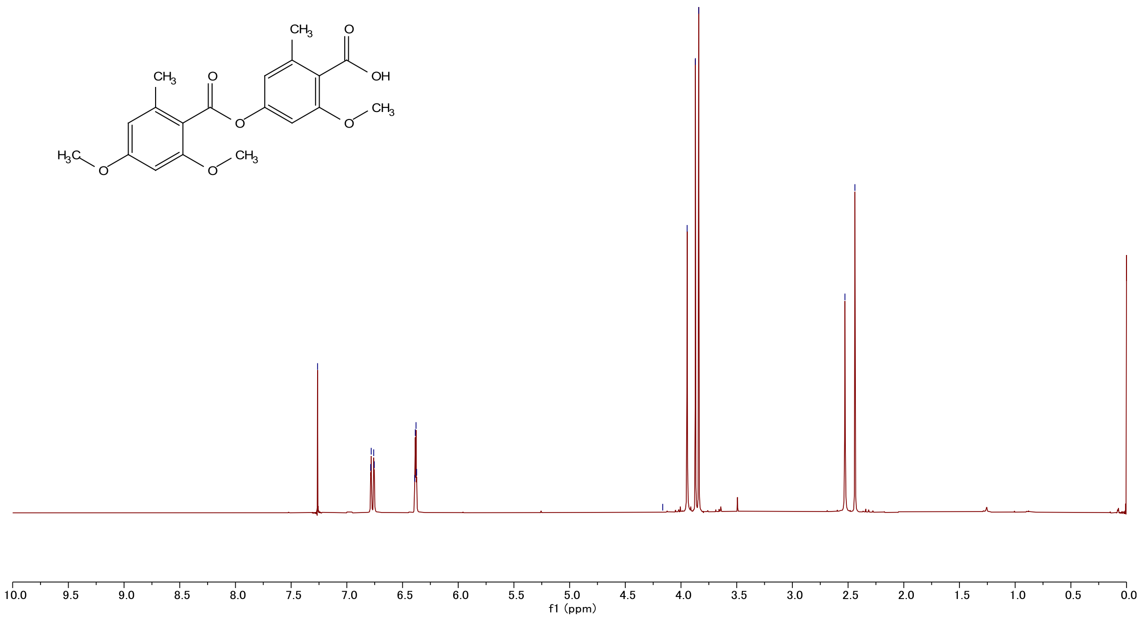

S1. ^{1}H NMR spectrum of compound **1** in $CDCl_3$

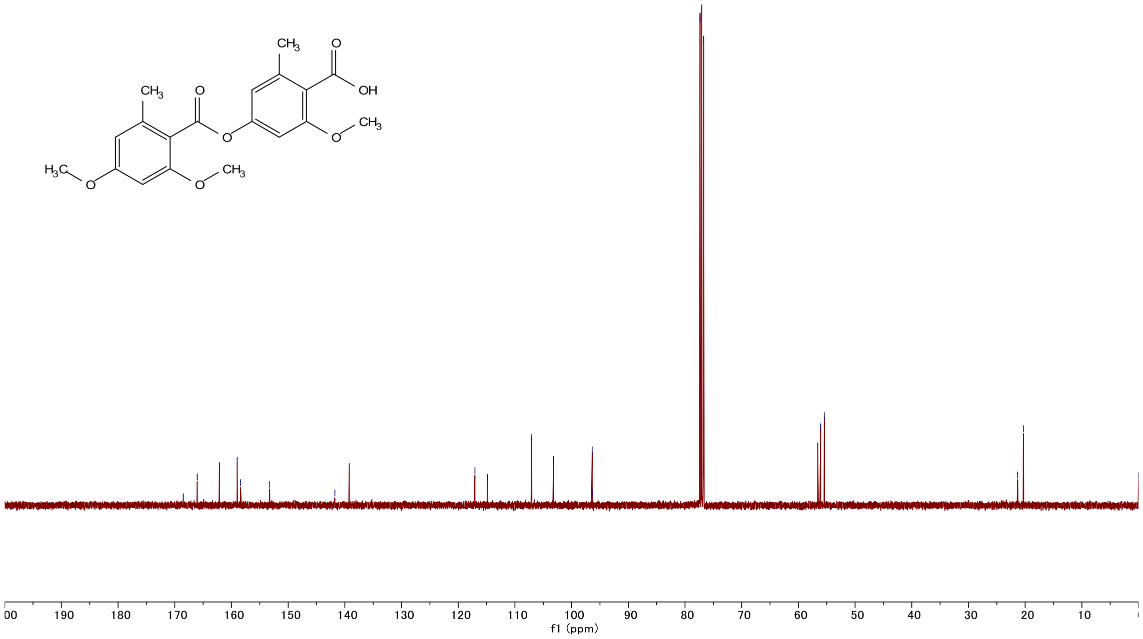

S2. ^{13}C NMR spectrum of compound **1** in $CDCl_3$

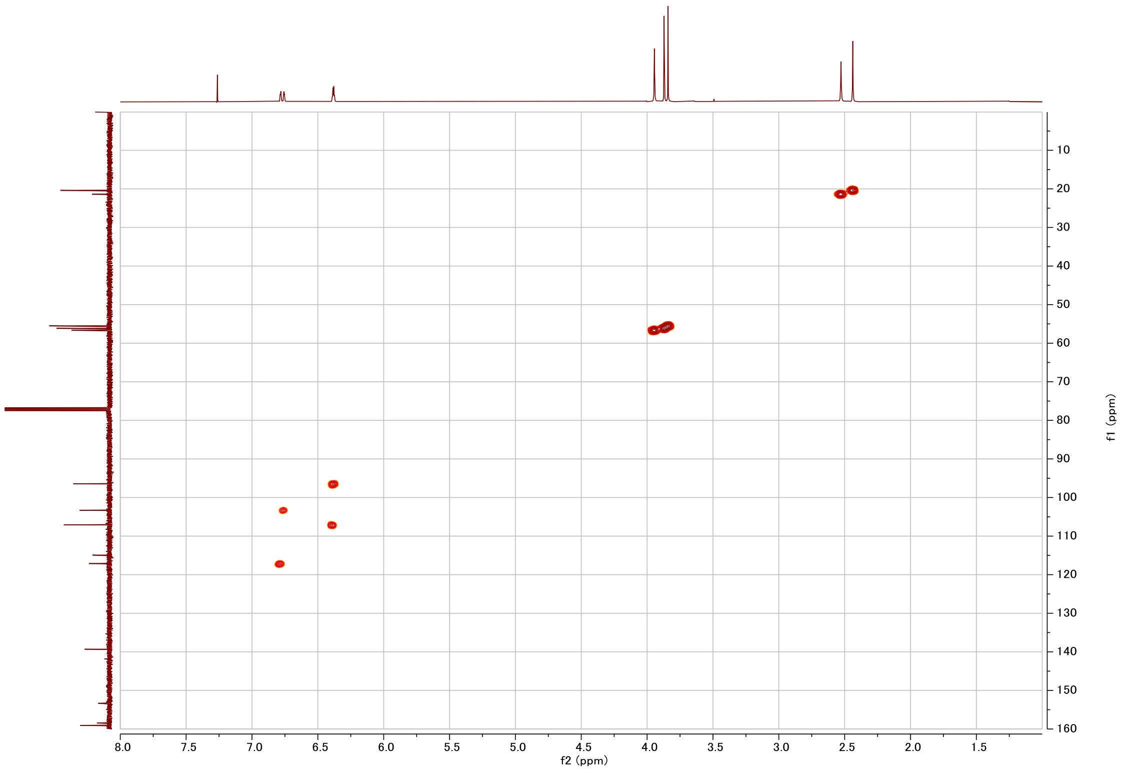

S3. HMQC spectrum of compound **1** in $CDCl_3$

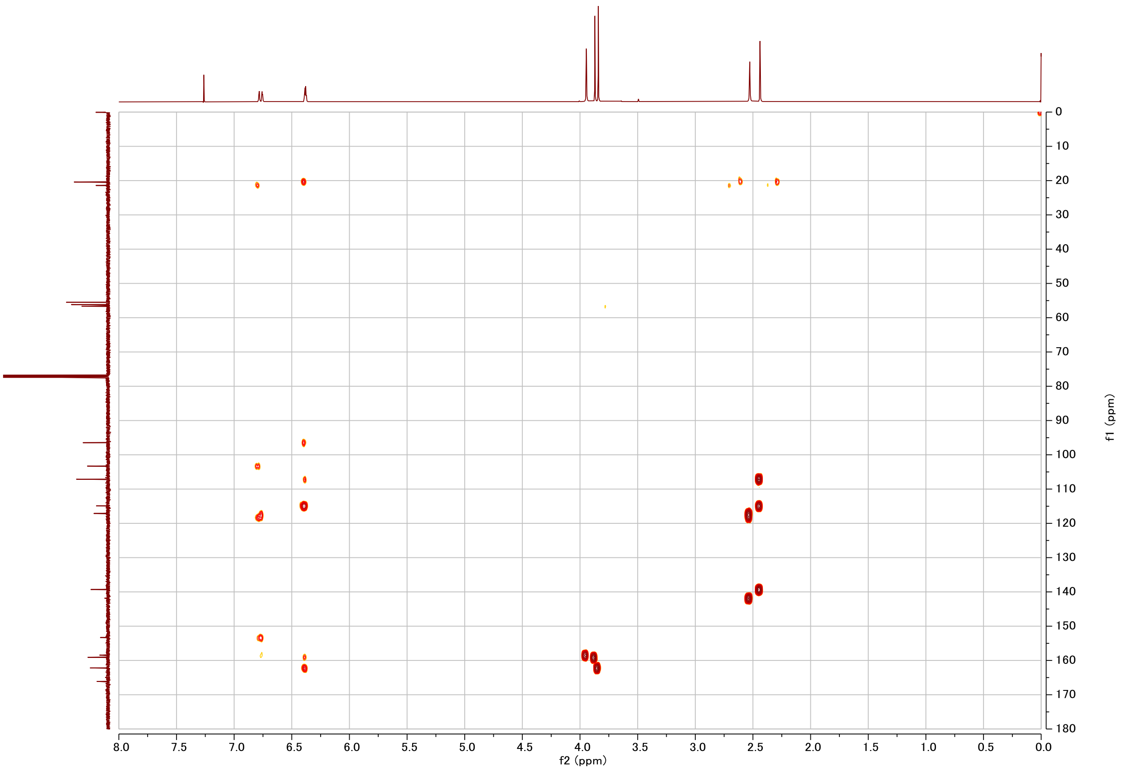

S4. HMBC spectrum of compound **1** in $CDCl_3$

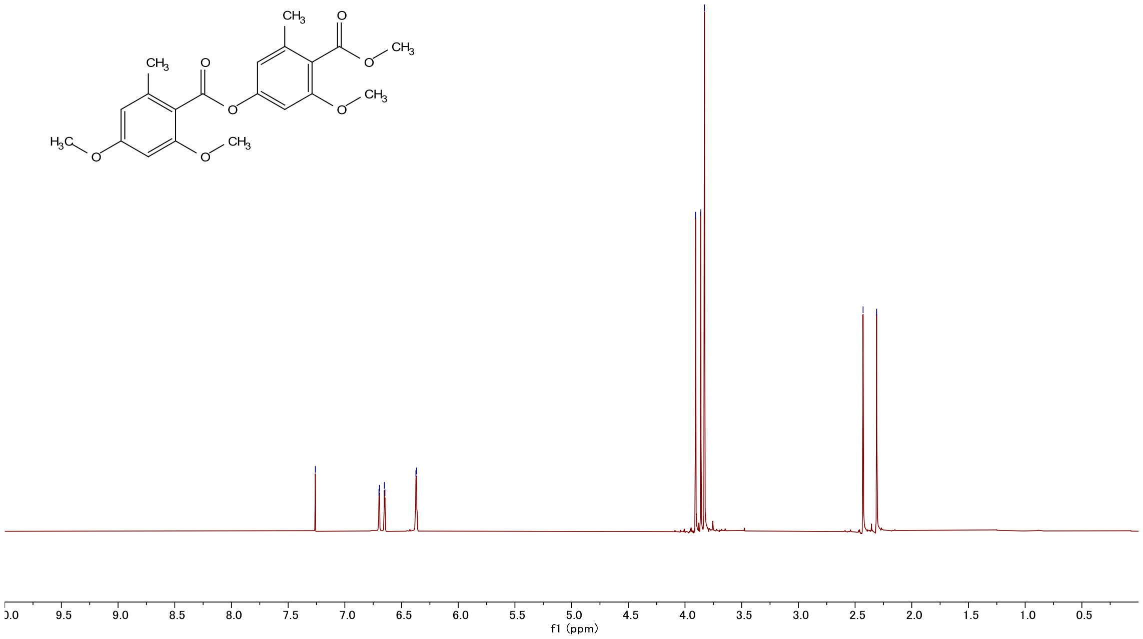

S5. ^{1}H NMR spectrum of compound **2** in $CDCl_3$

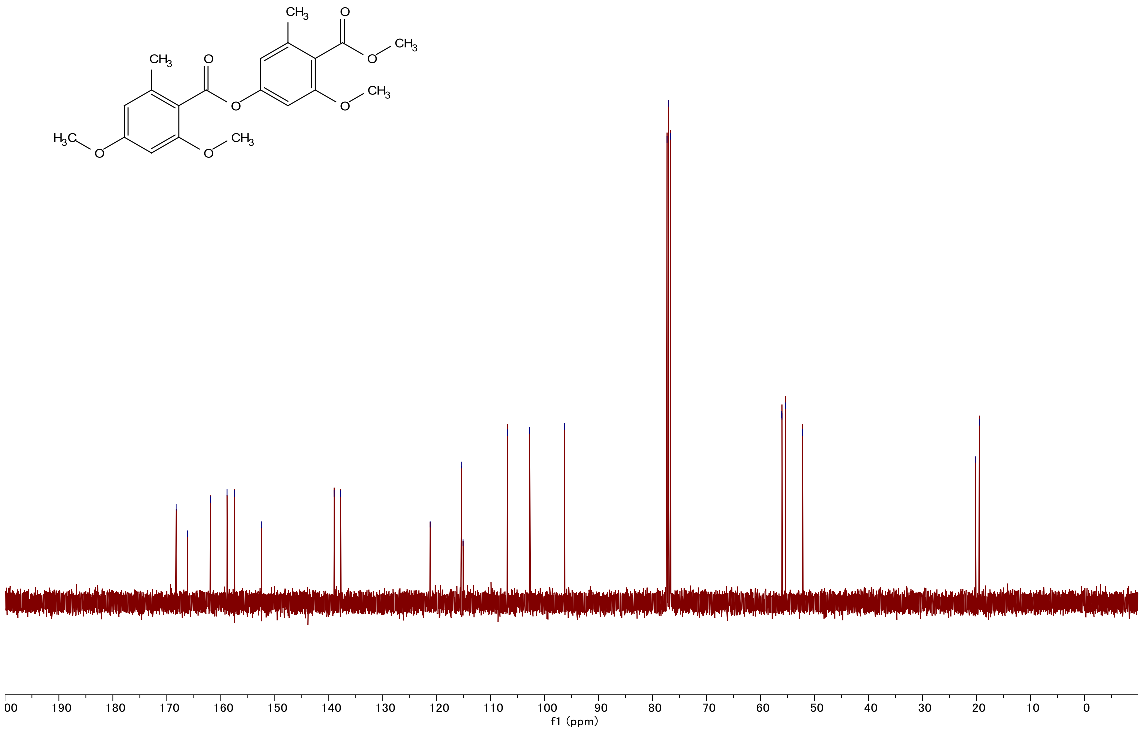

S6. ^{13}C NMR spectrum of compound **2** in $CDCl_3$

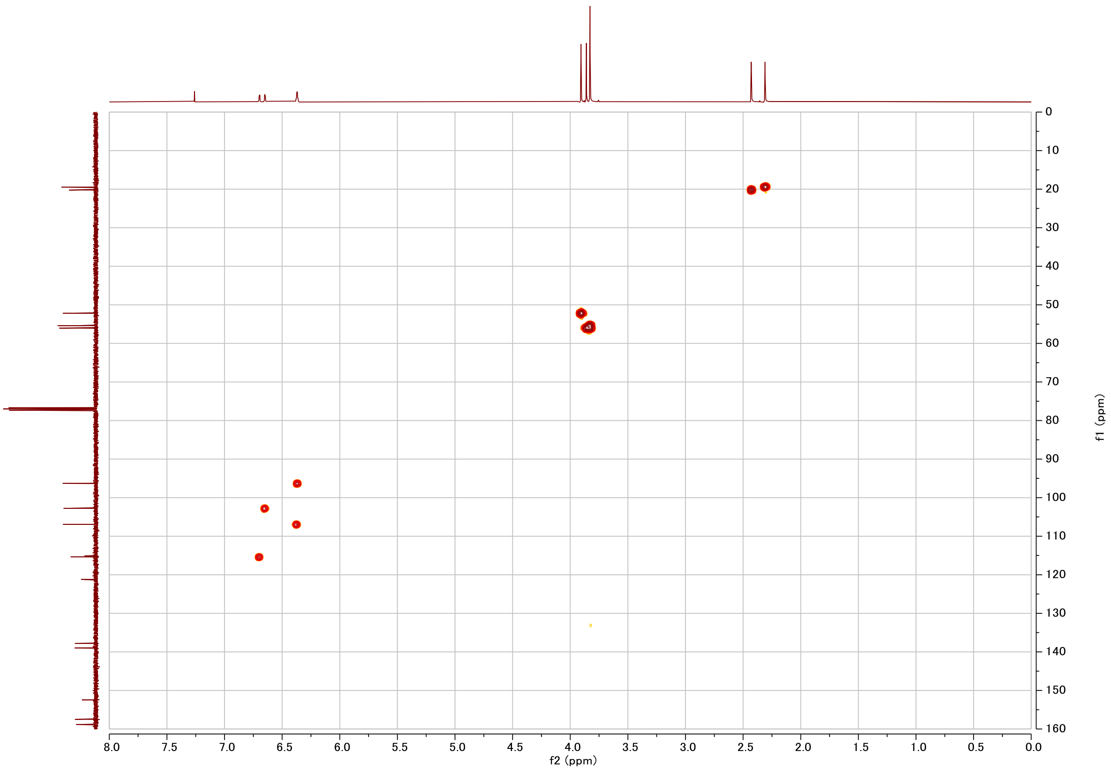

S7. HMQC spectrum of compound **2** in $CDCl_3$

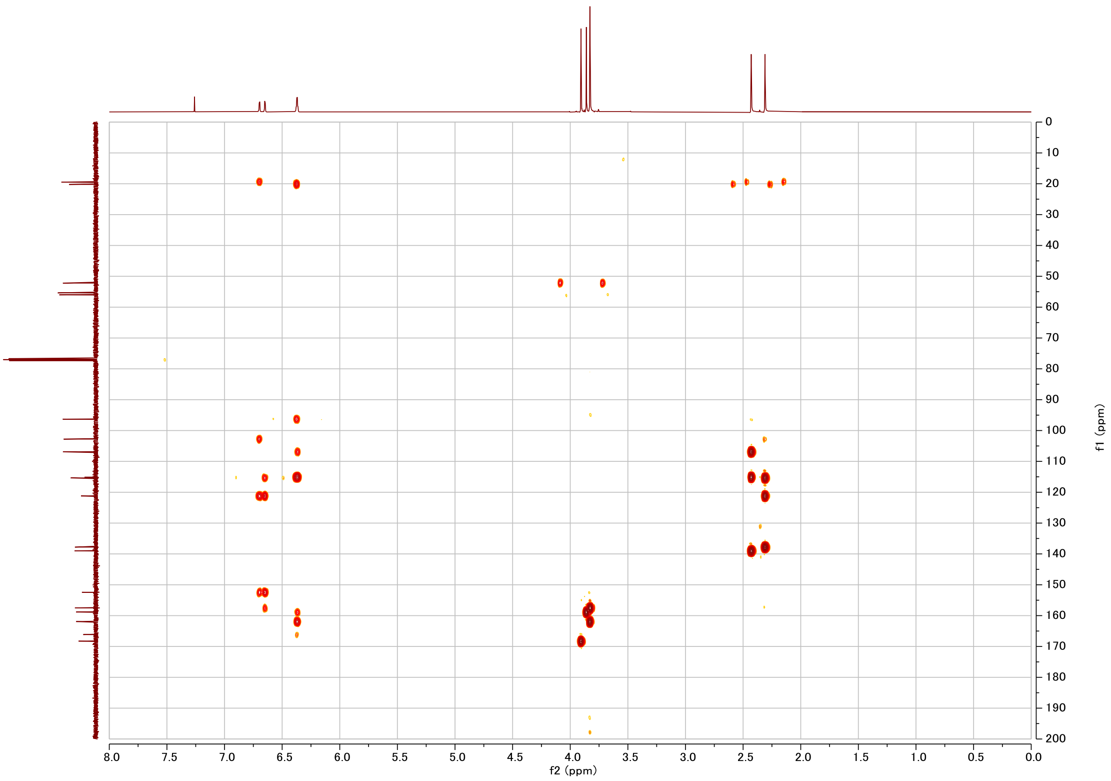

S8. HMBC spectrum of compound **2** in $CDCl_3$

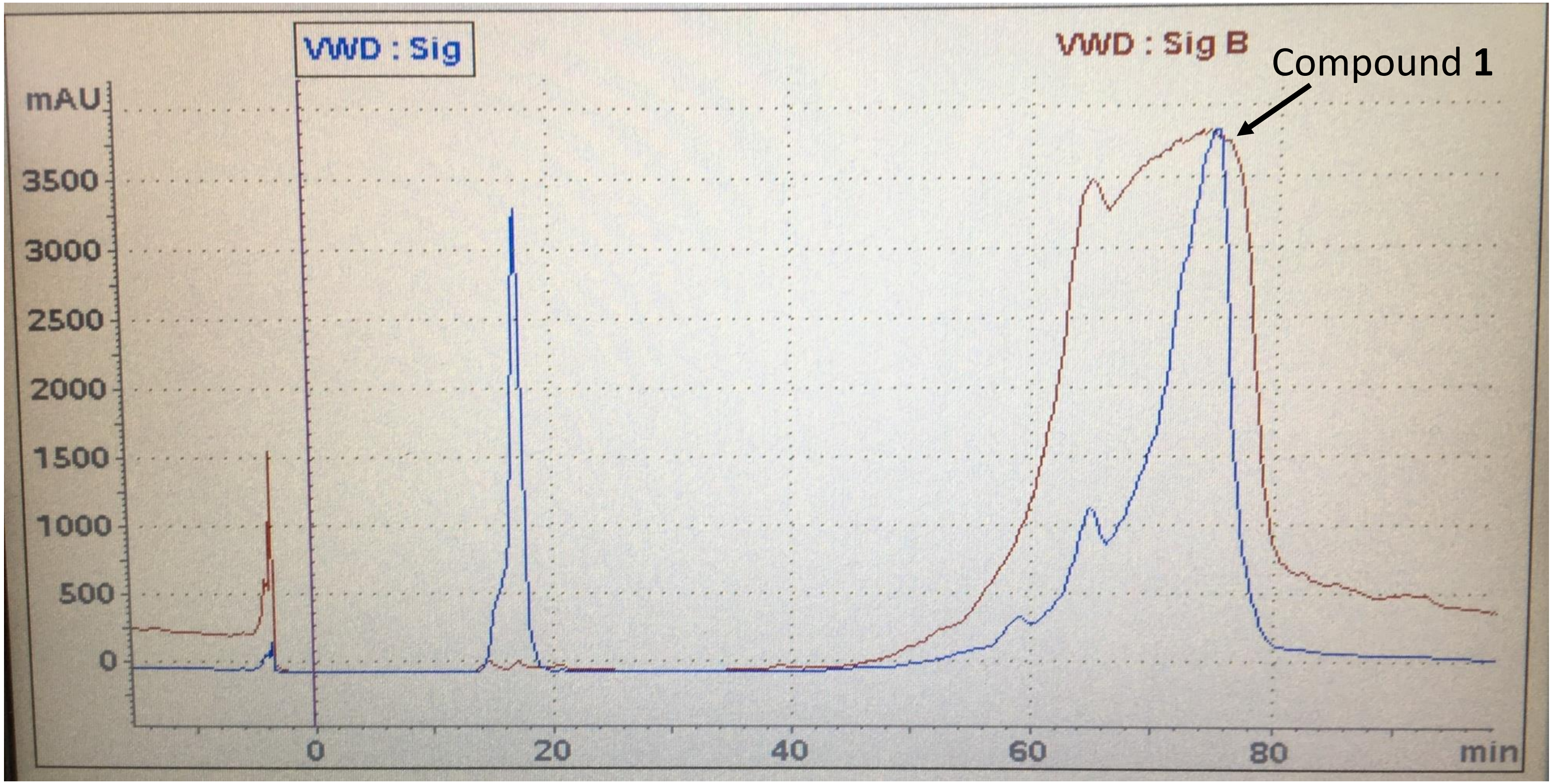

S9. HPLC chromatogram for compound **1** purification (solvent used methanol 50%, UV 254 nm (blue line) and 210 nm (red line), flow rate 4 mL/min.

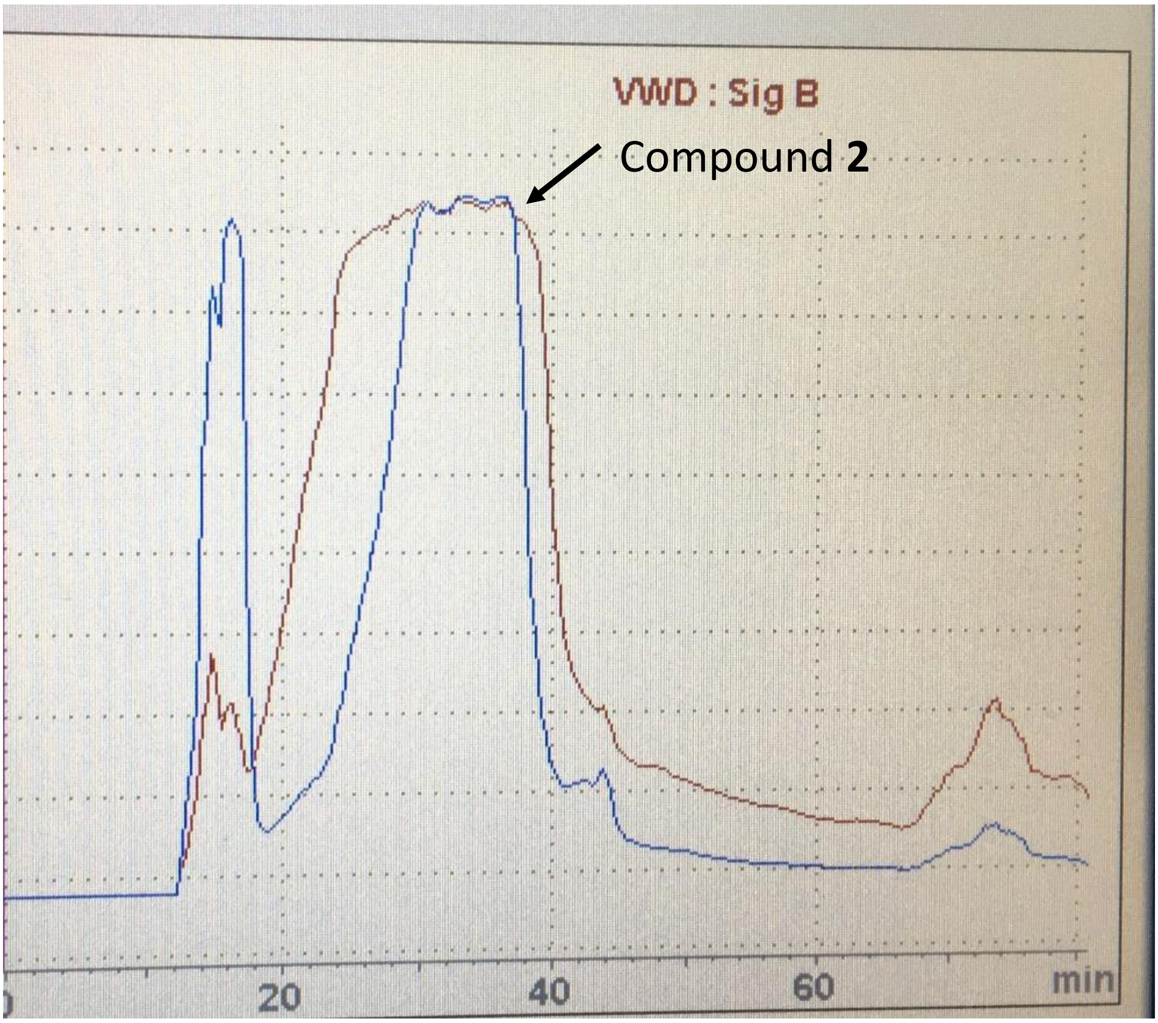

S10. HPLC chromatogram for compound **2** purification (solvent used methanol 70%, UV 254 nm (blue line) and 210 nm (red line), flow rate 4 mL/min.

S11. Virtual Similarity Screening and Target Prediction Methodology for compounds 1 and 2

Virtual similarity screening for compounds 1 and 2

The SwissSimilarity program (http://www.swisssimilarity.ch/) was used to virtually screen compounds **1** and **2** against a list of bioactive chemicals from the Ligand Expo database [6]. Fingerprint FP2 (FP2), a path-based fingerprint in the Open Babel chemical toolbox, was utilized for the 2D similarity search [10]. SwissSimilarity also uses a "combined" 2D/3D screening method for the reverse screening algorithm of the SwissTargetPrediction web application. The likelihood that two bioactive chemicals to share a protein target is represented by the calculated similarity score which is computed using logistic regression according to FP2 and Electropshape-5D (ES5D) similarity scores.

Target prediction for compounds 1 and 2

SwissTargetPrediction determines the degree of similarity between compounds **1** and **2** with those found in well selected, purified sets of well-established active ingredients in precise experimental binding tests. There are two ways to quantify similarity which involve calculating a pairwise comparison of 1D vectors that describe molecular structures in both cases; a 2D measure using the Tanimoto index between FP2 and a 3D measure based on a Manhattan distance similarity quantity between ES5D float vectors, mining five characteristics (partial charge, lipophilic contribution, and Cartesian coordinates) for every atom of 20 previously created conformations. The idea is that two molecules that are similar are represented by equivalent vectors that have a quantitative similarity near 1. This is true for both 2D and 3D similarity measurements. The SwissTargetPrediction model weights 2D and 3D similarity characteristics in a so-called Combined-Score by fitting a multiple logistic regression on different size-related subsets of known actives. The compounds are likely to share a protein target if the Combined-Score is greater than 0.5.
